# Lutetium‐Based Nanoprobes for Radiosensitization with Immune Microenvironment Remodeling and NIR‐II Fluorescence Imaging‐Guided Surgery in Colorectal Cancer

**DOI:** 10.1002/advs.202510136

**Published:** 2025-07-21

**Authors:** Yongying Dang, Xianzhi Liu, Zifan Zheng, Ao Wang, Ying Huang, Zhong Luo, Haina Tian, Siyaqi Li, Qiang Luo, Peiyuan Wang, Weiling He

**Affiliations:** ^1^ Department of Gastrointestinal Surgery Xiang'an Hospital of Xiamen University School of Medicine Xiamen University Xiamen 361100 P. R. China; ^2^ Key Laboratory of Design and Assembly of Functional Nanostructures Fujian Institute of Research on the Structure of Matter Chinese Academy of Sciences Fuzhou 350002 P. R. China; ^3^ Department of Etiology and Carcinogenesis National Cancer Center/National Clinical Research Center for Cancer/Cancer Hospital Chinese Academy of Medical Sciences and Peking Union Medical College Beijing 100021 P. R. China; ^4^ School of Chemistry and Materials Engineering & Engineering Research Center of Biomass Conversion and Pollution Prevention Control of Anhui Provincial Department of Education Fuyang Normal University Fuyang 236037 P. R. China

**Keywords:** colorectal cancer, immune microenvironment remodeling, NIR‐II fluorescence imaging, precise discrimination of surgical margins, radiotherapy sensitization

## Abstract

Colorectal cancer (CRC) is among the top five leading cancers worldwide. Preoperative concurrent chemoradiotherapy is recommended for locally advanced CRC. Radiotherapy (RT), a traditional cancer treatment, not only controls local tumor growth but also potentially induces immunogenic cell death, initiating systemic immune responses. Given the poor radiosensitivity of CRC, improving RT sensitization is a critical unmet need. Despite advances in intraoperative imaging, achieving complete resection of colorectal tumors with clear margins in real time remains a significant clinical challenge. This study introduces RVLu@ICG, a novel multifunctional fluorescent nanoprobe emitting in the second near‐infrared (NIR‐II) range. It's demonstrated that RVLu@ICG has tumor‐specific targeting due to modification with cyclic arginine‐glycine‐aspartic acid (c(RGDfK)) pentapeptide and induces augmented reactive oxygen species (ROS) production under ionizing radiation exposure. This synergistic mechanism not only potentiates radiosensitization efficacy but also facilitates radiation‐induced remodeling of the tumor immune microenvironment. Additionally, NIR‐II fluorescence image guidance facilitates precise surgical navigation in microtumor models, intramuscular tumor invasion models, and peritoneal metastasis models. Notably, the nanoprobe demonstrates excellent biocompatibility both in vitro and in vivo. Thus, RVLu@ICG establishes a robust precision therapy platform for the treatment of CRC.

## Graphical abstract

Enhancing radiosensitization, modulating the tumor immune microenvironment, and ensuring complete resection are crucial for colorectal cancer (CRC) treatment. This study presents a multifunctional NIR‐II fluorescent nanoprobe, RVLu@ICG, which enhances radiosensitization, remodels the tumor immune microenvironment, and enables precise image‐guided resection in CRC models. Demonstrating excellent biocompatibility, RVLu@ICG offers a promising platform for integrated radio‐immunotherapy and surgical management of CRC.

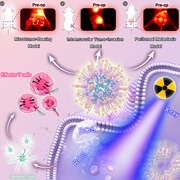

## 1 Introduction

According to Global Cancer Statistics 2022, colorectal cancer (CRC) ranks among the most prevalent malignancies globally, constituting 9.6% of total cancer diagnoses and 9.3% of cancer‐related mortality.^[^ ^1^ ^]^ Neoadjuvant radiotherapy or chemoradiotherapy (RT/CRT) represents a clinically recommended neoadjuvant therapeutic regimen for patients with locally advanced CRC. This intervention achieves the reduction of tumor volume to enable surgical resection, particularly for locally advanced or anatomically constrained lesions.^[^ ^2^ ^]^ Emerging evidence indicates that, beyond direct tumoricidal effects, RT exerts pleiotropic immunomodulatory effects on the tumor microenvironment (TME). Notably, ionizing radiation promotes the presentation of tumor‐associated antigens (TAAs) and triggers the release of damage‐associated molecular patterns (DAMPs) in the TME.^[^ ^3^, ^4^ ^]^ This process facilitates their recognition by dendritic cells (DCs)‐expressed pattern recognition receptors, thereby initiating a signaling cascade culminating in immunogenic cell death (ICD) of malignant cells.^[^ ^5^, ^6^ ^]^

Due to the moderate radiosensitivity of CRC and the anoxic microenvironment of the tumor, higher and potentially harmful radiation doses are often required to achieve adequate tumoricidal effects.^[^ ^7^ ^]^ Paradoxically, the therapeutic intensification may negatively impact normal tissues by disrupting vascular permeability, inducing fibrosis, and worsening hypoxia.^[^ ^8^, ^9^ ^]^ An effective strategy to address these limitations involves augmenting the therapeutic efficacy of radiotherapy (RT) while concurrently amplifying RT‐induced ICD through the incorporation of radiosensitizing agents within the TME. High atomic number (Z) elements—such as gold,^[^ ^10^ ^]^ selenium,^[^ ^11^ ^]^ and gadolinium,^[^ ^12^ ^]^—exhibit promising radiosensitizing properties by enhancing X‐ray absorption and retention. Upon irradiation, these metals emit secondary electrons that facilitate intracellular water radiolysis, leading to the generation of free radicals capable of inducing DNA double‐strand breaks (DSBs), with reduced off‐target toxicity.^[^ ^13^ ^]^ Rare earth based nanoparticles have been widely proven to be effective as radiation sensitizers. Compared to the elements reported above, the atomic number of lutetium (Lu) is higher (Z = 71), exhibiting greater sensitivity to RT. This fundamental property endows Lu‐based nanosensitizers with superior translational potential in precision RT, particularly for deep‐seated malignancies requiring optimized radiation dose deposition.

Emerging evidence suggests that the efficacy of radiosensitization arises from its capacity to remodel the TME, augment cellular susceptibility to ionizing radiation, and potentiate antitumor immune activation. However, these strategies remain insufficient for complete eradication of tumor tissues. Consequently, radical surgical resection remains a core component in the treatment of locally advanced CRC. By surgically removing all visible tumor tissue and ensuring negative margins (R0 resection), the risks of postoperative local recurrence and distant metastasis can be significantly reduced, thereby improving patients' long‐term survival rates.^[^ ^14^ ^]^ And the intraoperative determination of tumor margins currently relies primarily on pathological diagnosis through frozen section analysis.^[^ ^15^ ^]^ However, this analysis could not provide real‐time intraoperative identification of margins, and limited sampling points could neither comprehensively reflect the whole tumor margin.^[^ ^16^ ^]^ Approximately 5% of colorectal cancer (CRC) cases present with positive surgical margins, a rate that significantly increases with tumor progression—reaching up to 28% in cases of locally advanced disease—thereby contributing to elevated recurrence risk.^[^ ^17^, ^18^, ^19^ ^]^ Additionally, peritoneal metastases (PM) occur in an estimated 3–20% of CRC patients, further complicating clinical outcomes.^[^ ^20^ ^]^

Encouragingly, fluorescence‐based imaging offers non‐invasive and repeatable in vivo visualization, delivering real‐time feedback with exceptional temporal and spatial resolution. It's expected to achieve the task of differentiating malignant tissue from benign tissue.^[^ ^21^, ^22^ ^]^ Over the last few years, FDA‐approved near‐infrared regtion (NIR, 650–1700 nm) fluorophore indocyanine green (ICG) has been extensively investigated for image‐guided surgery within the second near‐infrared (NIR‐II, 1000–1700 nm) window due to its favorable optical properties. NIR‐II offers less tissue absorption, autofluorescence, and photon scattering, deeper tissue penetration, better spatial resolution, and an improved tumor‐to‐background ratio (TBR).^[^ ^23^, ^24^, ^25^, ^26^ ^]^ But concerns remain regarding ICG's limited anti‐photobleaching and tumor tissue recognition capabilities.^[^ ^27^, ^28^ ^]^ Fortunately, our previous work demonstrated that loading ICG into the mesopores of nanocarriers significantly enhances its photostability performance.^[^ ^29^ ^]^ Consequently, the design of mesoporous nanocarriers capable of responding to the TME for ICG loading presents a promising strategy for enabling NIR‐II fluorescence‐guided surgical interventions.

Molecular image‐guided cancer therapy has gained considerable attention as a promising strategy for enhancing treatment outcomes.^[^ ^30^ ^]^ This approach enables accurate tumor localization and facilitates sustained in vivo accumulation of therapeutic agents. In the pursuit of more efficient image‐guided modalities, the development of multifunctional therapeutic agents has become a focal point. Various inorganic nanomaterials—such as iron oxide‐based nanoparticles, gold nanostructures,^[^ ^31^ ^]^ gold nanostructures,^[^ ^32^ ^]^ and Gadolinium‐Bismuth composites^[^ ^33^ ^]^—have been investigated. Despite their potential, clinical translation of many of these nanoplatforms remains limited due to concerns over biostability, biocompatibility, and synthetic complexity. Notably, our previous work introduced a degradable, virus‐mimetic hollow nanoparticle based on neodymium (Nd), which disassembles into ∼5 nm Nd_2_O_3_ nanogranules under physiological conditions. Remarkably, approximately 40% of these nanoprobes are renally cleared within 24 hours, highlighting their favorable in vivo safety profile.

Additionally, we found the unique virus‐like mesoporous exhibited a higher and faster internalization rate than ordinary mesoporous silica nanoparticles.^[^ ^34^, ^35^ ^]^ Therefore, we strategically incorporated ICG into a biodegradable Lu_2_O_3_ nanoprobe with hollow virus‐like morphology (VLu@ICG). Specially, since α_v_β_3_ integrin was overexpressed in CRC, we conjugated tumor‐targeting peptide, cyclic arginine‐glycine‐aspartic acid (c(RGDfK)) motifs (RVLu@ICG), on the prepared novel Lu‐based virus‐like mesoporous RT sensitizer, to pursue specific binding to α_v_β_3_ integrin receptors to improve the precise targeting capability of CRC.

**Figure 1:**
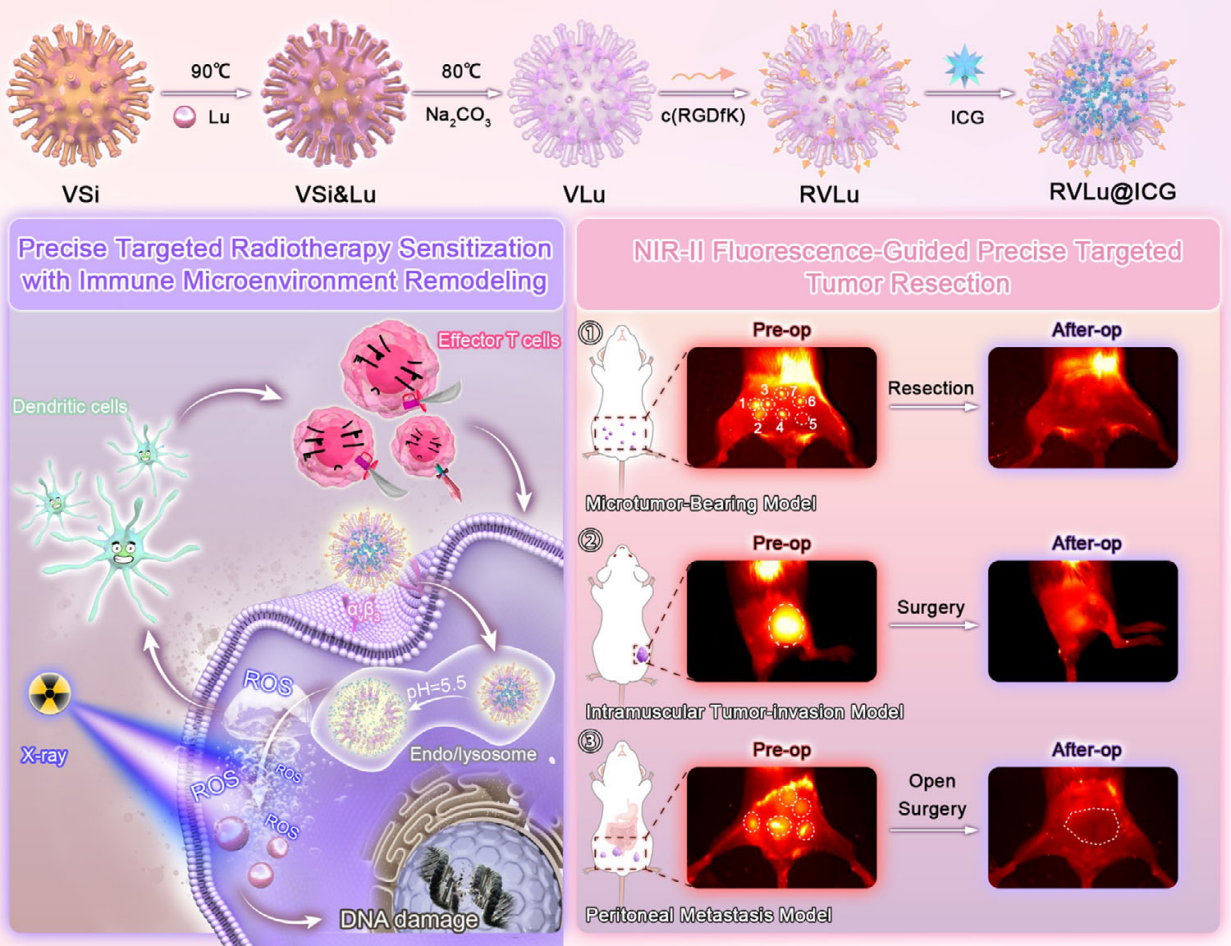
Schematic representation of the synthesis and application of RVLu@ICG nanoprobe for NIR‐II fluorescence‐guided targeted radiosensitization, modulation of the immune microenvironment, and precise CRC surgical resection. Abbreviations: VSi, virion‐like mesoporous silica nanoparticle; Pre‐op, preoperative; After‐op, postoperative.

Finally, we successfully developed a multifunctional nanoreagent RVLu@ICG for RT enhancement, immune microenvironment remodeling, and fluorescence imaging (**Figure** **1**). CRC can be thoroughly suppressed by the highly effective RT and immune activation both in vitro and in vivo. What's more, residual tumor tissues were successfully excised under NIR‐II fluorescence guidance, with negligible evidence of local recurrence or distant metastasis observed within 28 days postoperatively. Collectively, these findings underscore the promising preclinical utility of a NIR‐II biodegradable radiosensitizer in improving therapeutic outcomes through efficacious RT, immune microenvironment remodeling, and enhancing precise tumor margin detection.

## 2 Results

### 2.1 Fabrication and Physicochemical Profiling of RVLu@ICG

**Figure 2:**
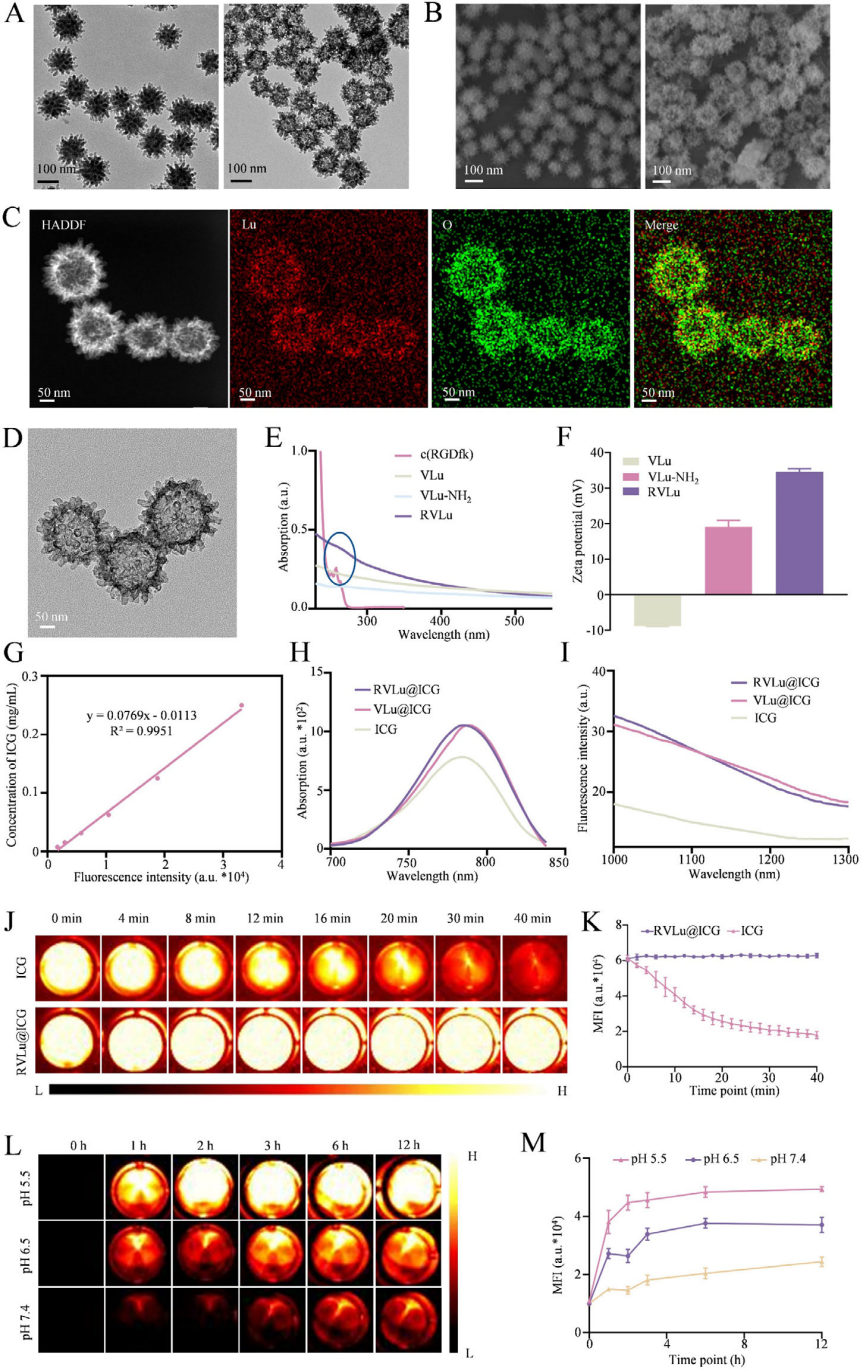
Synthesis and characterization of the nanoprobe. A) TEM micrographs of VSi (left) and VLu (right). B) SEM images of VSi (left) and VLu (right). C) Elemental distribution mapping of VLu: left—HAADF image; middle—elemental maps showing Lu (red) and O (green); right—merged overlay. D) High‐resolution TEM of RVLu. E) UV–vis absorption spectra of c(RGDfK), VLu, VLu‐NH₂, and RVLu; blue circled regions highlight the distinctive absorption peak at 258 nm corresponding to the c(RGDfK) peptide. F) Zeta potential measurements of VLu, VLu‐NH₂, and RVLu (n = 3; data presented as mean ± SD). G) Standard curve for ICG quantification. H) Absorption spectra of free ICG, VLu@ICG, and RVLu@ICG. I) Fluorescence emission profiles within the NIR‐II window for ICG, VLu@ICG, and RVLu@ICG. J) NIR‐II fluorescence images of ICG (top) and RVLu@ICG (bottom) under continuous 808 nm laser excitation (0.1 W/cm^2^) at 37 °C over time. K) Quantitative fluorescence intensity corresponding to panel (J). L) NIR‐II fluorescence images of RVLu@ICG supernatants incubated at pH 7.4, 6.5, and 5.5 for varied time intervals. M) Fluorescence intensity analysis corresponding to panel L). Abbreviation: HAADF, high‐angle annular dark‐field imaging.

Virion‐mimicking mesoporous silica nanoparticles (VSi) were fabricated via a conventional biphasic synthesis utilizing a hard‐template methodology. After 72 hours of mild agitation in a slightly alkaline environment, transmission electron microscopy (TEM) revealed the successful generation of uniform ≈100 nm particles with distinct virus‐like surface architecture (**Figure** **2**A). The VSi structure served as the core scaffold, while Lu(NO_3_)_3_·6H_2_O was employed as the Lu source and urotropine acted as the reductant. After a 17‐hour reaction at 80/90 °C, homogeneous hollow virion‐like mesoporous Lu_2_O_3_ nanospheres (VLu) with a diameter of ≈110 nm was successfully obtained, displaying a rough surface morphology (Figure 2A). Scanning electron microscopy (SEM) further revealed that both VSi and VLu exhibited a uniform biomimetic virus‐like nanostructure (Figure 2B). High‐angle annular dark field (HAADF) scanning electron microscopy, accompanied by elemental mapping, demonstrated a uniform spatial distribution of Lu and oxygen (O) within individual VLu nanostructures, verifying the effective construction of the Lu‐integrated hollow nanoprobes (Figure 2C).

VLu nanospheres were subsequently functionalized with amino groups using amino silane (3‐aminopropyl) triethoxysilane (APTES). To achieve covalent conjugation of c(RGDfK) peptides onto the VLu surface (RVLu), a standard N‐hydroxysuccinimide (NHS)/1‐(3‐dimethylaminopropyl)‐3‐ethylcarbodiimide hydrochloride (EDC) reaction was performed. As illustrated in Figure S3 (Supporting Information), RVLu retained the original surface morphology of VLu. High‐resolution transmission electron microscopy (HRTEM) analysis further confirmed that RVLu consisted of interpenetrated nanogranules (Figure 2D), while the absence of lattice fringes indicated their amorphous nature. ″Moreover, the UV‐vis absorption spectrum revealed a distinct absorption peak at 258 nm, characteristic of the c(RGDfK) peptide, verifying its successful attachment to the nanoshell surface (Figure 2E). Based on the standard calibration curves of c(RGDfK) (Figures S4 and S5, Supporting Information), the c(RGDfK) loading was quantified to be 17.01%, corresponding to a mass ratio of 1:11.76 (c(RGDfK) to RVLu@ICG, w/w). Moreover, zeta potential results showed that the surface charge varied during different preparation stages, again implying the successful construction of RVLu (Figure 2F). Furthermore, dynamic light scattering (DLS) measurements revealed that the hydrodynamic diameters of VLu, VLu‐NH_2_, and RVLu particles were 112.45 nm, 120.54 nm, and 136.87 nm, respectively, thereby validating the effectiveness of the peptide conjugation strategy (Figure S6, Supporting Information).

Subsequently, ICG was encapsulated within the hollow RVLu particles (RVLu@ICG), with an ICG loading efficiency of 23.56% according to the ICG standard curve (Figure 2G). Absorption spectra revealed a prominent peak near 780 nm for both RVLu@ICG and free ICG, indicating successful encapsulation of ICG within the nanoprobe cavity (Figure 2H). Moreover, upon 808 nm laser excitation and detection through a 1000 nm long‐pass filter, both formulations displayed characteristic NIR‐II emission profiles exceeding 1000 nm, affirming their optical activity in NIR‐II window (Figure 2I). Collectively, we have successfully engineered RVLu@ICG for acting a NIR‐II contrast agent. And the mole ratio of ICG, Lu and c(RGDfK) in RVLu@ICG was figured out by UV‐vis spectrophotometer and inductively coupled plasma as 1:330.65:18.54.

The significant photobleaching of ICG has greatly limited its clinical utility. To evaluate its stability in comparison to ICG, we investigated the photostability of RVLu@ICG dispersed in PBS. As depicted in Figure 2J,K, following continuous irradiation with an 808 nm laser at room temperature, RVLu@ICG owned a superior photobleaching resistance with approximately 100% fluorescence intensity maintaining, while that of ICG only maintained 28.74%. Because ICG is unstable in aqueous solutions, the NIR‐II fluorescence intensity of ICG and RVLu@ICG (dispersed in PBS for different time at room temperature) were latterly measured. As a result, free ICG lost approximately 84.27% of its initial fluorescence intensity over 12 hours. In contrast, RVLu@ICG retained more than 75% of its initial fluorescence intensity after 12 hours (Figures S8 and S9, Supporting Information). These findings clearly indicate that the aqueous stability of ICG was significantly enhanced when encapsulated in RVLu. The fluorescence results collectively confirmed the potential of RVLu@ICG as a NIR‐II contrast agent for prolonged surgical navigation under laser irradiation. As we reported previously,^[^ ^34^ ^]^ these distinctive hollow virus‐like nanoparticles were formed through the surface assembly of metal oxide nanogranules, indicating that RVLu possessed pH‐sensitive decomposition performance. As displayed in Figure S10 (Supporting Information), the nanoprobe gradually degraded in a normal physiological environment (pH 7.4), maintaining relatively structural stability for up to 36 hours. In contrast, RVLu demonstrated progressive degradation over time under acidic conditions representative of the intracellular milieu (pH 5.5) and the TME (pH 6.5), achieving complete decomposition within 6 and 12 hours, respectively. As anticipated, a large number of Lu‐containing nanogranules with diameters of < 5 nm were observed by high‐resolution TEM (HRTEM) following a 6‐hour incubation at pH 5.5 (Figure S11, Supporting Information), suggesting that the interparticle interactions within RVLu nanoparticles become destabilized under mildly acidic conditions, resulting in structural disassembly. Notably, the resulting small nanogranules are rapidly excreted via renal metabolism.^[^ ^35^ ^]^ To evaluate the pH‐responsive release behavior of ICG from RVLu@ICG under varying physiological and acidic conditions (pH 7.4, 6.5, and 5.5), release kinetics were systematically assessed. As depicted in Figure 2L,M and Figure S12 (Supporting Information), incubation at pH 7.4 for 12 hours resulted in the liberation of less than 35% of the encapsulated ICG, indicating minimal leakage under neutral conditions. In contrast, a significant release of ICG from RVLu@ICG was observed when the pH was reduced to 6.5. Within 2 hours of incubation at pH 5.0, the release of ICG reached up to 90%. These results proved that our TME RVLu@ICG facilitated the rapid diffusion of ICG within the tumor and demonstrated very high biological safety.

### 2.2 Assessment of Cancer Cell‐Specific Targeting Efficiency of RVLu@ICG in Vitro

**Figure 3:**
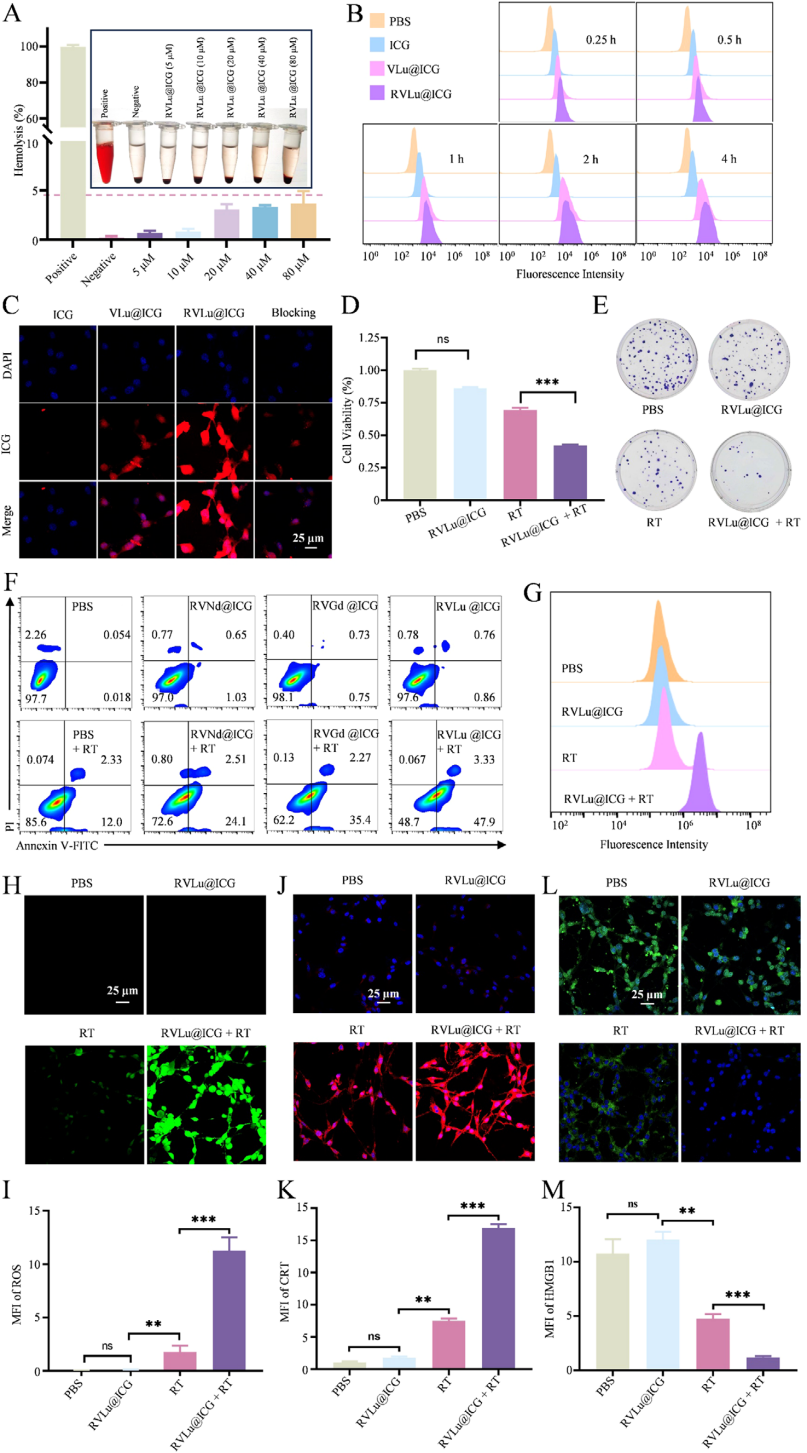
Cell cytotoxicity, cellular uptake and RT sensitivity of RVLu@ICG in vitro. A) Representative images and quantitative analysis of hemolysis for various treatments (n = 3). B) Flow cytometry quantification of CT26 cell internalization of ICG, VLu@ICG, and RVLu@ICG at 0.25, 0.5, 1, 2, and 4 hours post‐incubation. C) CLSM images of CT26 cells after 2‐hour incubation with ICG, VLu@ICG, RVLu@ICG, and RVLu@ICG following c(RGDfK) pretreatment. D) Viability of CT26 cells treated with PBS or RVLu@ICG, with or without 4 Gy X‐ray irradiation; non‐irradiated PBS and RVLu@ICG served as controls (n = 3). E) Colony formation assays of CT26 cells exposed to various formulations. F) Apoptosis analysis by flow cytometry in CT26 cells treated with PBS, RVNd@ICG, RVGd@ICG, and RVLu@ICG under 4 Gy irradiation; corresponding non‐irradiated groups served as controls (n = 3). G) Intracellular ROS levels measured by flow cytometry in CT26 cells after PBS or RVLu@ICG treatment with/without 4 Gy X‐ray exposure. H, I) CLSM images and MFI analysis of ROS levels in CT26 cells following different treatments. J, K) CLSM images and MFI analysis of calreticulin (CRT) surface exposure in CT26 cells following different treatments. L, M) CLSM visualization and MFI quantification of HMGB1 release from treated CT26 cells. Scale bars represent 25 µm. Statistical significance was determined by two‐way ANOVA and two‐tailed unpaired t‐tests: **P < 0.01; ***P < 0.001; ****P < 0.0001. Data are presented as mean ± SD (n = 3).

Cancer cell precise recognition is essential to precise treatment of tumor tissues successfully. RVLu@ICG was utilized for tumor cell targeting studies using flow cytometry and confocal laser scanning microscopy (CLSM). Prior to assessing cellular internalization, the biocompatibility of the nanoprobes was evaluated through hemolysis and cell proliferation assays. The results from the hemolysis experiment (**Figure** **3**A) show no significant hemolysis, with the hemolysis ratio of RVLu@ICG at all concentrations (0–80 µM) remaining below 5%, indicating good blood compatibility. Additionally, as shown in Figure S13 (Supporting Information), the viability of CT26 cells remained above 87% after incubation with RVLu@ICG (0‐40 µM), suggesting that RVLu@ICG exhibits no obvious toxicity of RVLu@ICG.

Meanwhile, the cellular uptake efficiency of the CT26 cell line was conducted after treatment with different formulations of nanoprobes. According to previous reports, the unique virus‐like spikes on the surface of nanoparticles can enhance cellular uptake,^[^ ^36^, ^37^ ^]^ possibly because these protrusions impose greater mechanical stress on the cell membrane compared to smooth‐surfaced particles, thereby strengthening their interaction with biological systems.^[^ ^38^ ^]^ To validate this hypothesis, we have successfully fabricated ≈110 nm normal mesoporous Lu_2_O_3_&NPs (MLu@ICG) (Figure S14, Supporting Information). As anticipated, the distinct virus‐like mesoporous structure of VLu@ICG demonstrated a significantly higher internalization rate compared to MLu@ICG, highlighting its superior cellular uptake efficiency. This enhanced recognition capability suggests improved targeting of cancerous tissues (Figure S14, Supporting Information). Later, we were encouraged to evaluate the CT26 tumor recognition ability of the biodegradable NIR‐II nanoprobes. Flow cytometry results demonstrated that RVLu@ICG achieved approximately twice the cellular uptake compared to VLu@ICG lacking c(RGDfK) functionalization (Figure 3B; Figure S15, Supporting Information), with a statistically significant difference (*P* < 0.001). Finally, the endocytosis of CT26 cells was further performed by CLSM. After a 4‐hour incubation with tumor cells, the RVLu@ICG group exhibited significantly higher red fluorescence (ICG) intensity, with a mean fluorescence intensity (MFI) three times stronger than that of the VLu@ICG group (Figure 3C; Figure S16, Supporting Information; *P* < 0.01). These findings validate the improved cellular uptake and selective tumor‐targeting properties of the nanoprobes conferred by c(RGDfK) functionalization. Additionally, a competitive inhibition assay was performed by pre‐incubating cells with an excess of c(RGDfK) peptide. In this pre‐treated group, RVLu@ICG uptake was notably reduced, as evidenced by both flow cytometry analysis (Figures S17 and S18, Supporting Information; *P* < 0.001) and CLSM imaging (Figure 3C; Figure S16, Supporting Information; *P* < 0.0001). These findings further validate that the Lu‐based nanoprobes functionalized with c(RGDfK) can accurately recognize and target tumor cells.

### 2.3 Radiation Sensitization in Vitro

Subsequently, we were inspired to evaluate the sensitizing effect of RVLu@ICG in RT. Under X‐ray irradiation (4 Gy), the cytotoxicity of nanoparticles (Lu^3^⁺ concentration = 20 µM) was notably higher than that of radiation alone, indicating that nanoparticle‐mediated RT significantly increased radiation‐induced cell death in tumor cells (*P* < 0.001) (Figure 3D). Additionally, colony formation assays were performed to evaluate the prolonged radiation sensitization effect of RVLu@ICG. CT26 tumor cells treated with RT and nanoparticles formed fewer viable cell colonies compared to the RT group, with a significant difference (*P* < 0.001) (Figure 3E; Figure S19, Supporting Information). Additionally, the impact of RVLu@ICG + RT on apoptosis was assessed. Flow cytometry analysis demonstrated a significantly higher apoptosis rate in the RVLu@ICG + RT group compared to RT alone (*P* < 0.001) (Figure 3F; Figure S21, Supporting Information). Moreover, the radiosensitization effect of Lu (RVLu@ICG) was markedly superior to that of Gd (RVGd@ICG) and Nd (RVNd@ICG) (Figure 3F; Figure S21, Supporting Information; *P* < 0.001). TEM analysis revealed that the morphologies of RVNd@ICG and RVGd@ICG were highly similar to that of RVLu@ICG (Figure S22, Supporting Information). This finding indicates that the observed differences in radiosensitizing efficacy are unlikely to result from morphological disparities, and are more likely associated with the higher atomic number of Lu. Further, we have also investigated the cancerous cell killing by RVLu@ICG + RT using a live/dead cell‐stained assay. Analogous to apoptosis findings, most of the tumor cells were stained with red fluorescent dyes when they were treated with RVLu@ICG + RT (Figure S23, Supporting Information). Ionizing radiation induces tumor cell apoptosis primarily via intracellular reactive oxygen species (ROS)‐mediated DNA damage.^[^ ^11^, ^39^ ^]^ To assess ROS levels, 2′,7′‐dichlorodihydrofluorescein diacetate (DCFH‐DA) staining was performed. Post‐irradiation, CT26 cells exposed to RVLu@ICG exhibited markedly enhanced green fluorescence relative to the PBS control, accompanied by a significant elevation in MFI (*P* < 0.0001) (Figure 3H,I; Figure S24, Supporting Information). Fluorescence signals from both the non‐irradiated PBS and RVLu@ICG groups were minimal (Figure 3H,I; Figure S24, Supporting Information). Flow cytometry results revealed that ROS levels in CT26 cells treated with RVLu@ICG combined with RT were approximately eightfold greater than those observed in the RT group (Figure 3G; Figure S25, Supporting Information), suggesting that RVLu@ICG significantly enhanced intracellular ROS production under X‐ray irradiation that substantially induced CRC cell apotosis/necrosis. These results further verified that RVLu@ICG possessed a powerful radiosensitization effect that exhibited significant impediments toward cancerous cell proliferation, and can significantly induce the death of cancer cells, making it a prospective radiotherapeutic nanoagent.

### 2.4 RT‐induced Immune Activation in Vitro

Extensive research has demonstrated that RT facilitates hydroxyl radical (•OH) generation upon ionizing radiation, thereby initiating ICD and stimulating antitumor immune responses. One hallmark of ICD is the translocation of calreticulin (CRT) from the endoplasmic reticulum to the plasma membrane. To assess this, immunofluorescence staining using Alexa Fluor 594‐conjugated anti‐CRT antibody was conducted. As depicted in Figure 3J,K, a significant increase in red fluorescence intensity was observed in CT26 cells following X‐ray exposure, indicating enhanced CRT surface expression. Notably, the RVLu@ICG + RT group exhibited even stronger CRT membrane localization compared to RT alone, attributable to the radiosensitizing effect of Lu. In parallel, the release of high mobility group box 1 (HMGB1), another key ICD marker, was investigated via immunofluorescence analysis. As ROS generated by RT compromise nuclear membrane integrity, HMGB1 is translocated to intercellular substance. Interestingly, an inverse trend was detected for HMGB1 relative to CRT expression (Figure 3L,M); the RVLu@ICG + RT group displayed the lowest intracellular green fluorescence signal among all treatments. Quantitative CLSM confirmed these findings, revealing that this combination therapy significantly reduced nuclear HMGB1 retention while promoting CRT externalization. Collectively, these results provide compelling evidence that RVLu@ICG, under X‐ray irradiation, induces robust ICD in vitro, thereby enhancing tumor immunogenicity and potentially contributing to a systemic (abscopal) antitumor response.

### 2.5 Biosafety, Cancer Targeting, and Biodistribution of RVLu@ICG in Vivo

**Figure 4:**
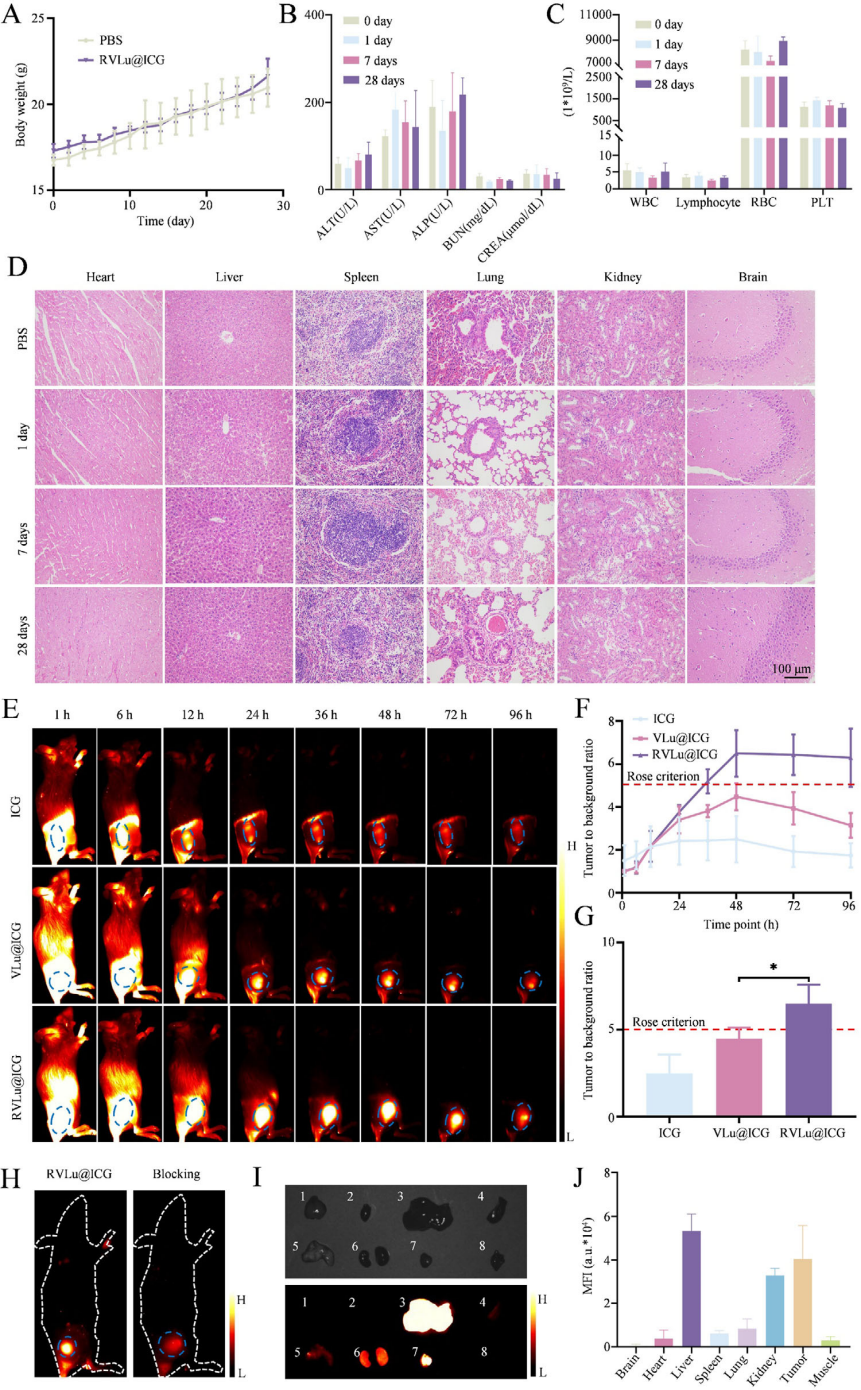
The safety and tumor targeting efficiency estimation of RVLu@ICG in vivo. A) Body weight monitoring of mice following intravenous administration of PBS or RVLu@ICG over 28 days. B) Biochemical blood panel analysis at multiple time points to assess hepatic and renal function after tail vein injection of RVLu@ICG (n = 3). C) Hematological profiles measured at different intervals post‐injection to evaluate effects on blood cell populations (n = 3). D) Representative HE stained sections of major organs from BALB/c mice after intravenous injection of PBS and RVLu@ICG for 1, 7, and 28 days, respectively (n = 3). E) Serial NIR‐II fluorescence imaging of mice following intravenous injection of ICG (top), VLu@ICG (middle), and RVLu@ICG (bottom) at various time points (n = 3). The blue dotted ellipses indicate the tumors. F) TBR quantification in tumor‐bearing mice injected with ICG, VLu@ICG, or RVLu@ICG over time (n = 3). G) Comparative analysis of tumor‐targeting efficiency among ICG, VLu@ICG, and RVLu@ICG at peak TBR. Statistical significance determined by two‐tailed unpaired t‐test: *P < 0.05; data expressed as mean ± SD (n = 3). H) Representative NIR‐II fluorescence images of the RVLu@ICG targeting group and c(RGDfK) blocking group (40 mg kg^−1^) at 48 hours post‐injection. The blue dotted ellipses indicate the tumors. I) *Ex vivo* bright‐field (top) and NIR‐II fluorescence images (bottom) of harvested organs (1‐8: brain, heart, liver, spleen, lung, kidney, tumor, muscle) collected 48 hours after RVLu@ICG administration. J) Quantification of MFI based on panel (I) (n = 3).

Before in vivo fluorescent imaging studies, in vivo safety was first investigated. Healthy female BALB/c mice were randomly assigned to four experimental groups. Similar to the PBS‐treated group, during the observation period of four weeks, no body weight alternation was recorded in the RVLu@ICG group (**Figure** **4**A). Venous blood samples were serially sampled at day = 0/1/7/28 for liver and kidney function assessments, along with routine hematological analysis. The data indicated stable levels of creatinine (CREA), alanine aminotransferase (ALT), aspartate aminotransferase (AST), and blood urea nitrogen (BUN) (Figure 4B). Furthermore, no notable changes were detected in platelet (PLT), red blood cell (RBC), lymphocyte (Lymph), or white blood cell (WBC) counts (Figure 4C). Histological examination of hematoxylin‐eosin (HE)‐stained organ sections revealed no apparent pathological changes in either group (Figure 4D). These findings suggest that RVLu@ICG exhibited minimal toxicity in mice, supporting its potential safety for future clinical applications.

To evaluate the in vivo targeting capability of RVLu@ICG, CT26‐Luc tumor cells were subcutaneously inoculated into BALB/c mice following the demonstration of efficient tumor cell identification in vitro and low systemic toxicity in vivo. Tumor‐bearing mice were administered ICG, VLu@ICG, or RVLu@ICG via the caudal vein. NIR‐II fluorescence imaging was conducted at various time intervals employing an 808 nm excitation wavelength (100 mW/cm^2^) alongside a 1000 nm long‐pass filter. As illustrated in Figure 4E, fluorescence signals in the tumor area were scarcely observable in the ICG‐treated group. In contrast, the RVLu@ICG group exhibited a strong tumor‐specific fluorescence signal, reaching its peak intensity 48 hours post‐injection (Figure 4F). Furthermore, the maximum TBR of the RVLu@ICG group (6.50 ± 1.10) was remarkably higher than the VLu@ICG group (4.49 ± 0.63) and ICG group (2.49 ± 1.08) (Figure 4G, *P* < 0.05). The fluorescence signals gradually diminished, becoming undetectable by 96 hours post‐injection. Significantly, the RVLu@ICG group exhibited sustained and elevated tumor fluorescence intensity compared to both VLu@ICG and ICG groups across all measured time points, underscoring the improved tumor‐targeting efficiency attributed to c(RGDfK) conjugation (Figure 4F). Based on the Rose criterion, a TBR exceeding 5 is considered adequate for precise tumor visualization.^[^ ^40^ ^]^ Thus, RVLu@ICG demonstrated superior tumor tissue discrimination compared to VLu@ICG and ICG. To further validate its targeting specificity, an in vivo blocking study was conducted by pre‐injecting c(RGDfK). Fluorescence imaging conducted 48 hours after administration demonstrated markedly greater signal intensity in the RVLu@ICG‐treated group relative to the receptor‐blocked control group (Figure 4H; Figure S26, Supporting Information; 7.65 ± 1.61 vs. 2.40 ± 0.44, *P* < 0.01). Collectively, these findings highlight the precise CRC‐targeting capability of RVLu@ICG. To further assess the biodistribution of the nanoprobe in vivo, mouse organs were harvested and subjected to fluorescence imaging 48 hours post‐injection, as illustrated in Figure 4I,J, these images and MFI revealed a high uptake in tumor as well as the reticuloendothelial system organs (including liver, kidney and lung).

### 2.6 Molecular Mechanism Analysis in Vivo

**Figure 5:**
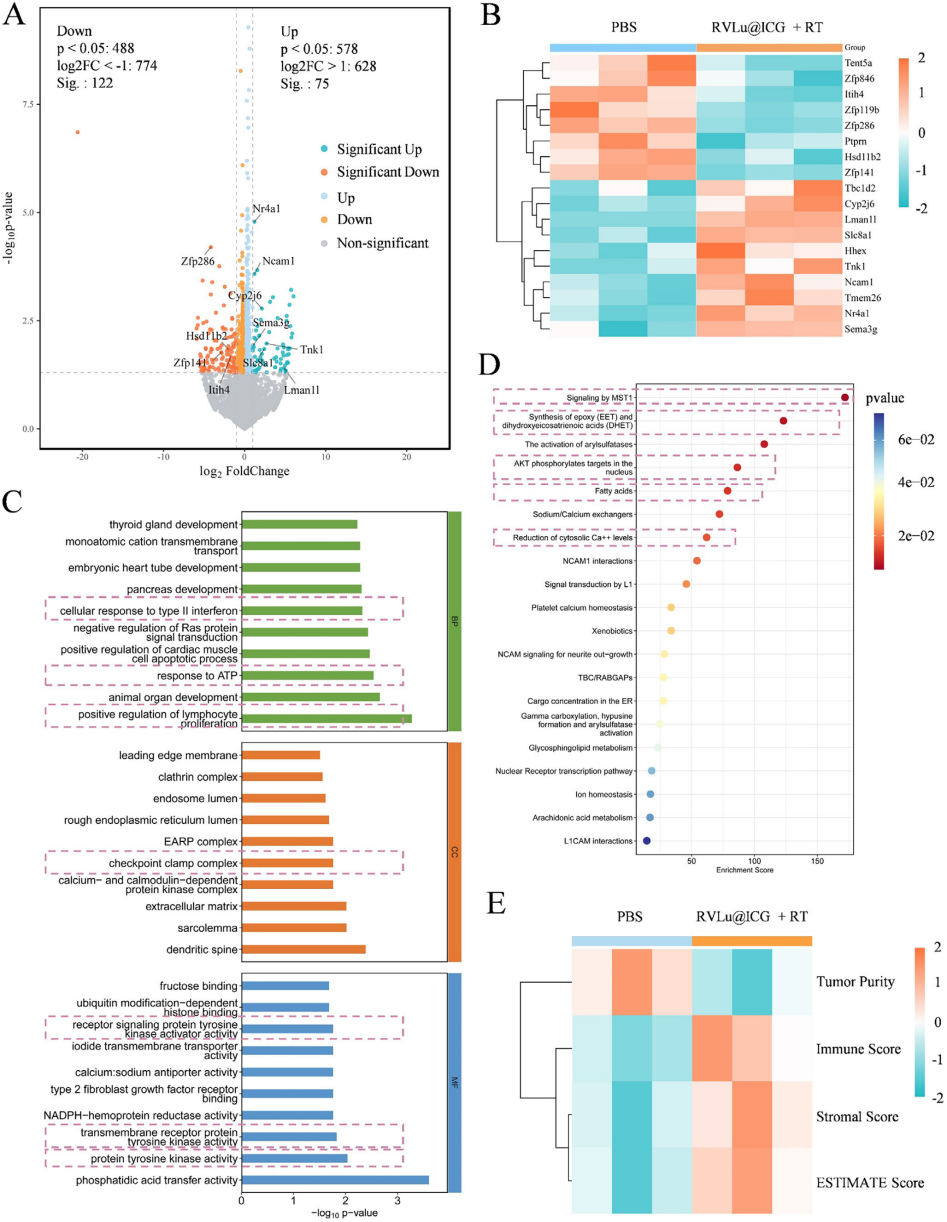
Molecular mechanism analysis of RVLu@ICG + RT in vivo. A) Volcano plot of differentially expressed genes. B) Clustering heatmap of individual genes in different treatment groups (Orange: up‐regulation; green: down‐regulation). C) Gene Ontology (GO) enrichment analysis of tumor tissues after receiving different treatments. D) Top 20 significantly enriched reactome pathways which were up‐regulated. E) Heatmap of TME ESTIMATE Scores in different treatment groups (Orange: up‐regulation; green: down‐regulation).

Having confirmed the excellent tumor targeting ability, we subsequently explored the potential biological effects and antitumor mechanisms of RVLu@ICG + RT from the RNA level. RNA sequencing (RNA‐seq) analysis of tumor tissues receiving PBS or RVLu@ICG + RT irradiation treatments was conducted to identify the differentially expressed genes and corresponding signaling pathways. Results indicated that RVLu@ICG + RT irradiation induced many genes expression changes, especially the upregulated expression of immune activation‐related genes (such as Slc8a1, Tnk1, Ncam1 and Nr4a1) and down‐regulated expression of immunosuppressive genes (such as Hsd11b2, Itih4, and Zfp286) (**Figure** **5**A,B), which posed impacts on the immune activation‐associated biological processes, cellular components, and molecular functions (Figure 5C). Correspondingly, the Reactome pathway enrichment analysis also uncovered significant up‐regulation of immune activation‐related pathways, such as signaling by MST1, AKT phosphorylates targets in the nucleus, arachidonic acid metabolism, and platelet calcium homeostasis (Figure 5D). Especially, the transmembrane receptor protein tyrosine kinase activity pathways were effectively activated, strongly implying the provoking of immune response during treatment of RVLu@ICG + RT irradiation (Figure S28, Supporting Information). Moreover, the Estimation of STromal and Immune cells in MAlignant Tumor tissues using Expression data (ESTIMATE) score heatmap indicates that the Immune Score in the RVLu@ICG + RT group is higher than that in the PBS group, suggesting that the RVLu@ICG + RT group has greater immune cell infiltration and stronger immune activity (Figure 5E). Given the above results, we could conclude that RVLu@ICG + RT irradiation possessed effective immunomodulation ability on the TME during therapy.

### 2.7 Radio‐Immunotherapy Antitumor Effect of RVLu@ICG in Vivo

**Figure 6:**
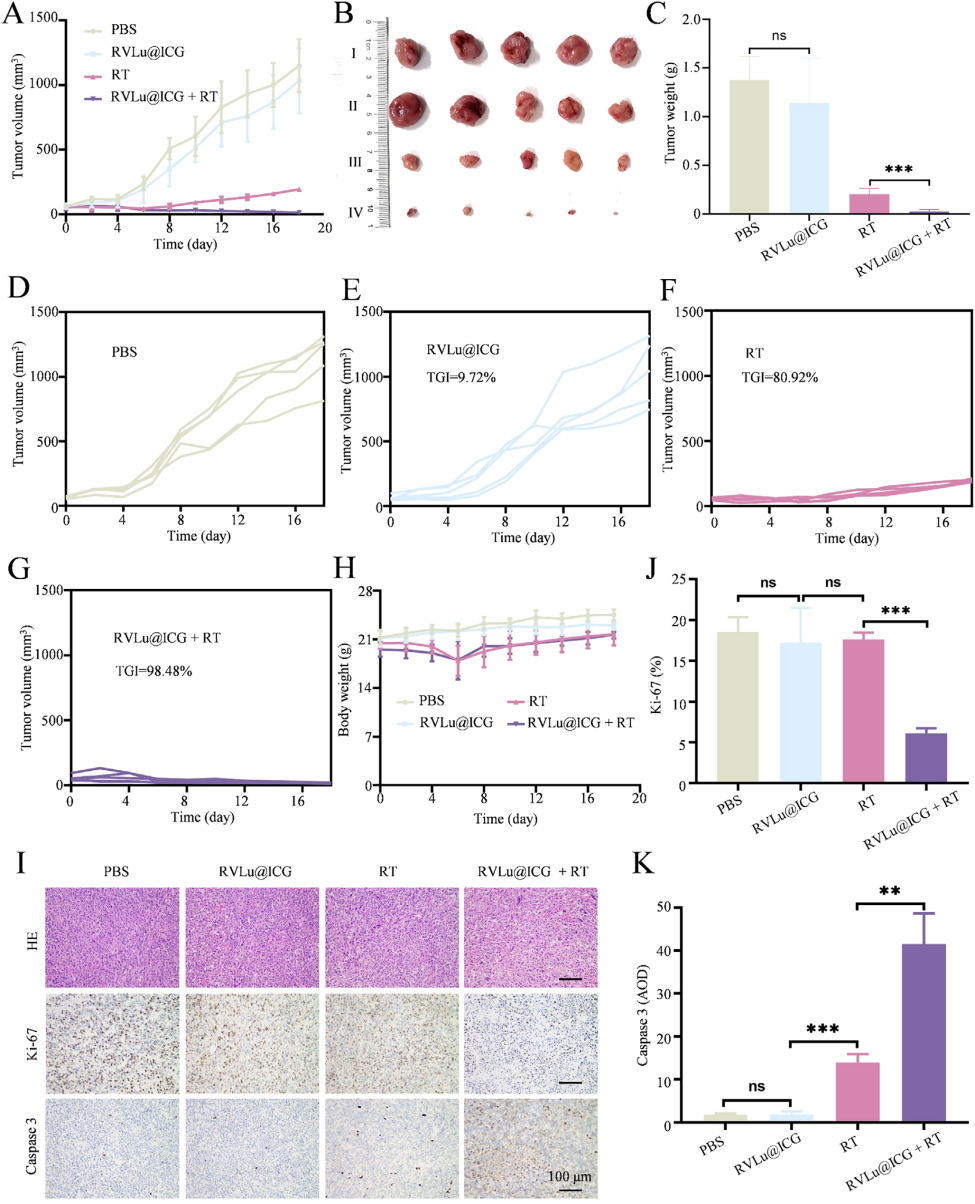
Radiosensitization of RVLu@ICG in CT26‐tumor‐bearing mice. A) Tumor growth curves of mice treated with PBS or RVLu@ICG, with or without 8 Gy X‐ray irradiation on day 2 (n = 5). B) Representative images of excised tumors from groups receiving PBS (I), RVLu@ICG (II), RT (III), and RVLu@ICG + RT (IV) treatments (n = 5). C) Tumor weights measured on day 18 post‐treatment (n = 5). Individual tumor volume progression for PBS D), RVLu@ICG E), RT F), and RVLu@ICG + RT G) groups (n = 5). H) Body weight monitoring of mice under different treatment regimens (n = 5). I) Representative histological staining of tumor sections for HE, Ki67 proliferation marker, and cleaved caspase‐3 apoptosis marker, collected 18 days after treatments (n = 3). Scale bars, 100 µm. J, K) Quantitative analysis of Ki67 and caspase‐3 positive cells from panel (I). Data are presented as mean ± SD. Statistical significance was determined by one‐way ANOVA with Tukey's post hoc test: *P < 0.05, **P < 0.01, ***P < 0.001.

Leveraging the X‐ray activated Compton scattering properties of Lu^3+^, tumor growth inhibition was evaluated in RVLu@ICG‐treated mice through intravenous administration. CT26 tumor‐bearing mice were randomly allocated into four groups: PBS, RVLu@ICG, RT, and RVLu@ICG + RT. Representative fluorescence images (Figure S29, Supporting Information) confirmed the probe's selective targeting of CRC, prompting further investigation into its role in RT. As depicted in **Figure** **6**A,D,E, tumor growth remained largely unrestrained in both nonirradiated PBS‐treated and RVLu@ICG‐treated mice. However, upon X‐ray exposure, RVLu@ICG‐treated mice exhibited significantly enhanced RT sensitization, leading to substantial tumor volume reduction compared to those receiving RT alone (Figure 6A,F,G). Furthermore, weight of resected tumors in RVLu@ICG + RT group is the lightest on day 18, confirmed substantial tumor elimination and growth suppression in RT‐sensitized animals compared to the other groups (Figure 6B,C). Six days post‐X‐ray radiation, the body weight of the mice slightly decreased but returned to normal levels by day 10 (Figure 6H).

To further evaluate the antitumor efficacy of radiotherapy, histological examination was conducted on tumor samples collected from various treatment groups after 18 days. Ki67 staining, a standard indicator of cellular proliferation, was utilized to assess in vivo therapeutic effects. Findings revealed a marked decrease in Ki67‐positive cells in the RVLu@ICG combined with RT group relative to all other cohorts (Figure 6I,J). Concurrently, caspase‐3 activity was markedly elevated in the RVLu@ICG + RT group relative to the remaining groups (Figure 6I,K). These findings indicate that RVLu@ICG + RT treatment effectively suppressed tumor cell proliferation while promoting apoptosis through the activation of effector caspase‐3 and its downstream pathways. Additionally, a TUNEL assay was conducted to assess apoptosis rates in tumor sections. Tumors exposed to radiation monotherapy exhibited only marginally increased apoptosis compared to those treated with PBS or RVLu@ICG alone. Conversely, the combination of RVLu@ICG with radiotherapy induced substantially elevated apoptosis rates relative to both PBS and RVLu@ICG groups (Figures S30 and S31, Supporting Information). These immunohistochemical data strongly indicate that RVLu@ICG combined with RT effectively enhances tumor cell apoptosis and suppresses tumor progression, corroborating the observations from HE staining analyses.

### 2.8 Immune Activation Analysis in Vivo

**Figure 7:**
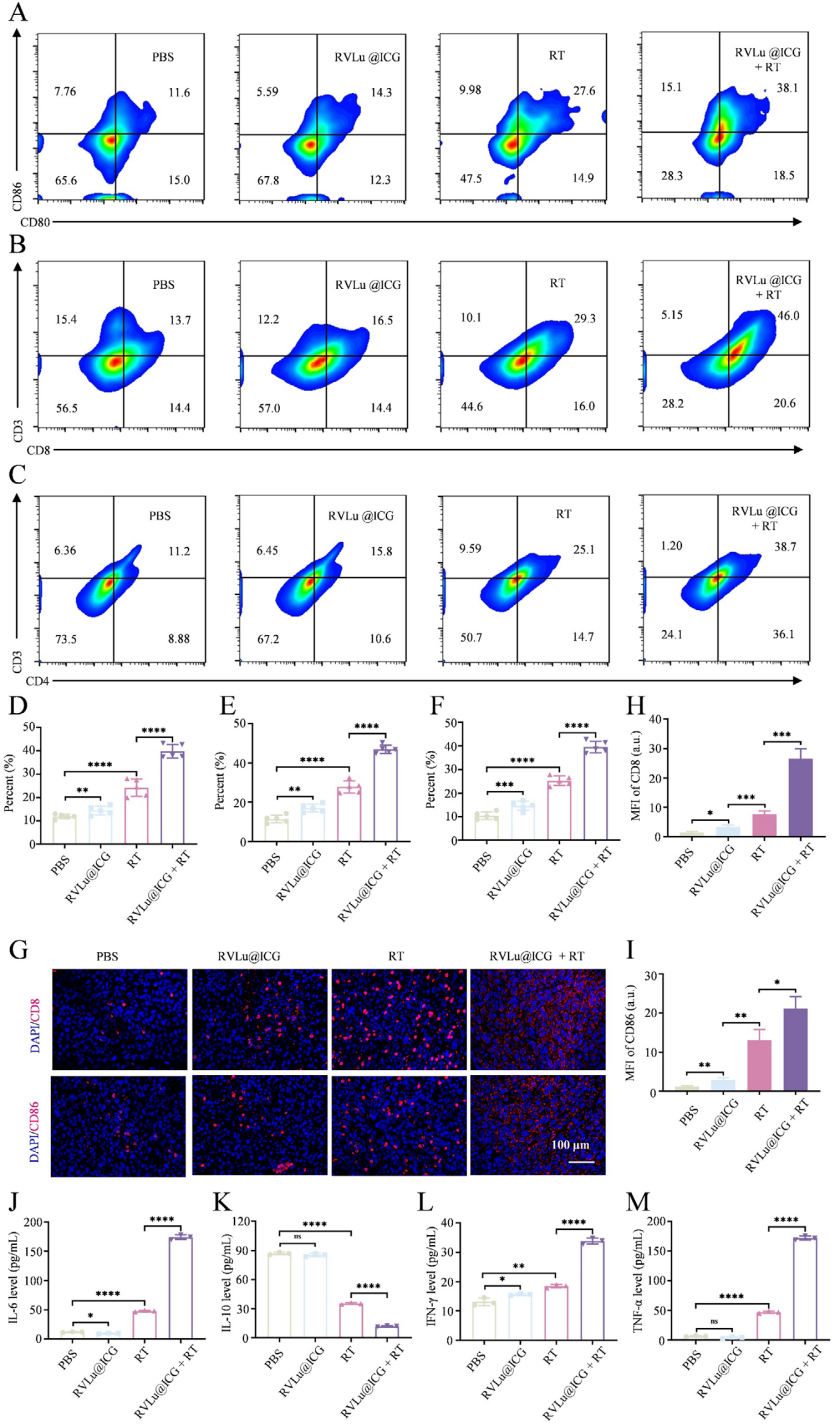
Immune activation analysis of RVLu@ICG + RT in vivo. A) Flow cytometry representative plots showing mature DCs (CD80^+^CD86^+^ gated on CD11c^+^) in tumor‐draining lymph nodes. B, C) Representative flow cytometric profiles of CD8^+^ (CD8^+^CD3^+^ gated on CD45^+^) and CD4^+^ (CD4^+^CD3^+^ gated on CD45^+^) T lymphocytes within tumor tissues. D–F) Quantitative analyses of mature DCs in lymph nodes, and CD8^+^ and CD4^+^ T cells in tumors across treatment groups (n = 5). G) Immunofluorescence images of tumor sections stained for nuclei (blue) and CD8^+^ or CD86^+^ cells (red) following various treatments. H, I) Quantification of MFI for CD8^+^ and CD86^+^ markers. J–M) ELISA measurements of serum cytokine concentrations for IL‐6, IL‐10, IFN‐γ, and TNF‐α post‐treatment (n = 3). Data are shown as mean ± SD. Statistical significance: **P < 0.01, ***P < 0.001, ****P < 0.0001.

The pronounced antitumor efficacy observed with RVLu@ICG + RT is largely attributable to the synergistic interaction between ionizing radiation and immune modulation. To further elucidate the mechanism of immune activation, we evaluated immune cell populations and cytokine expression levels using flow cytometry, immunofluorescence staining, and ELISA assays. Maturation of DCs within tumor‐draining lymph nodes was assessed, revealing elevated proportions of CD80⁺CD86⁺ mature DCs in both RT‐treated groups, with the RVLu@ICG + RT cohort exhibiting the most pronounced increase, indicative of enhanced antigen‐presenting activity (**Figure** **7**A,D; Figure S34, Supporting Information). Subsequent analysis of tumor‐infiltrating lymphocytes showed significantly increased frequencies of both CD8⁺CD3⁺ and CD4⁺CD3⁺ T cells in the RVLu@ICG + RT group, suggesting potent activation of cellular and adaptive immune responses (Figure 7B,C,E,F; Figure S35, Supporting Information). These findings were further corroborated by immunofluorescence imaging, which demonstrated a marked accumulation of CD8⁺ T cells and mature DCs within the tumor microenvironment (Figure 7G–I). Cytokine profiling further confirmed the immunostimulatory effect of the combined therapy. The RVLu@ICG + RT group exhibited the highest concentrations of proinflammatory cytokines—including IL‐6, IFN‐γ, and TNF‐α—alongside a notable suppression of the immunosuppressive cytokine IL‐10 (Figure 7J–M). Specifically, RT alone elevated IFN‐γ secretion compared to PBS and RVLu@ICG groups, confirming its role in inducing IFN‐γ production. The addition of RVLu@ICG to RT further augmented IFN‐γ levels, underscoring that RVLu@ICG can further amplify radiotherapy‐associated immune activation through its radiosensitizing effects. IFN‐γ as well as other immune response together confirmed that the RVLu@ICG could efficiently sensitizing RT and provoke potent antitumor immune response.

### 2.9 NIR‐II Fluorescence Image‐Guided Tumor Localization and Surgical Resection in a Microtumor Model

**Figure 8:**
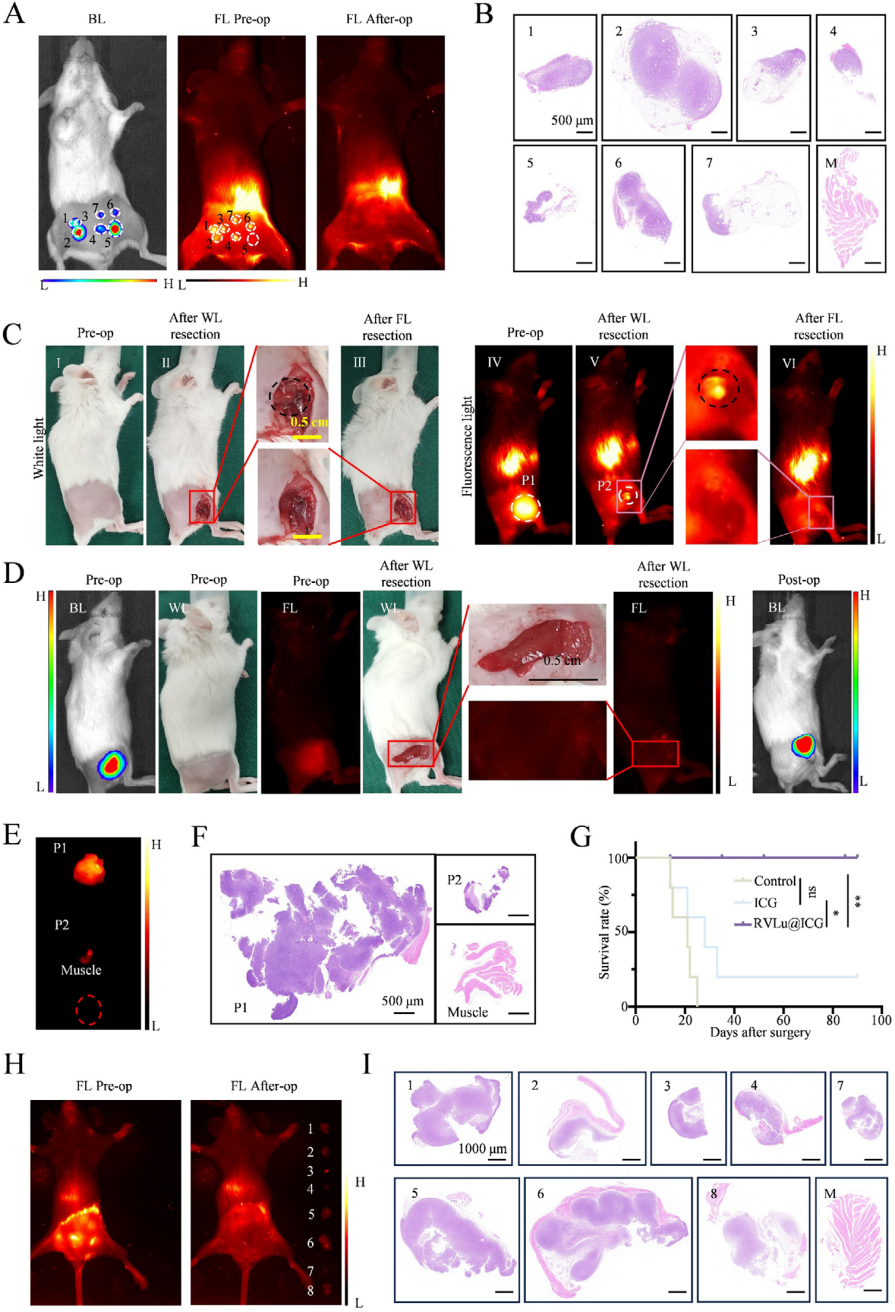
Surgery navigation of RVLu@ICG in the microtumor model, intramuscular tumor‐invasion model and the abdominal transfered carcinoma model. A) Representative preoperative bioluminescence, as well as pre‐ and post‐operative NIR‐II fluorescence images, of a mouse bearing multiple microtumors following RVLu@ICG injection. B) HE staining of tumor and adjacent muscle tissues corresponding to panel (A). C) Sequential imaging (white light and NIR‐II fluorescence) before, during, and after tumor excision in an intramuscular tumor‐infiltration model treated with RVLu@ICG (pre‐op: I, IV; intra‐op: II, V; post‐op: III, VI). D) Similar imaging of tumor resection guided by ICG in the same model, including bioluminescence, white light, and fluorescence views. E) Post‐surgical NIR‐II fluorescence images of two resected tumors (P1, P2) and adjacent muscle from panel (C). F) HE histology of excised tissues shown in panel (E). G) Kaplan–Meier survival analysis comparing outcomes among groups receiving RVLu@ICG‐, ICG‐, or white‐light‐guided surgery (n = 5); statistical significance determined using the Log‐rank test (*P* < 0.05). H) NIR‐II fluorescence‐guided pre‐ and post‐operative imaging of mice with peritoneally disseminated tumors. I) Corresponding HE‐stained sections of tumor and surrounding muscle. All scale bars represent 1000 µm.

The highest tumor‐to‐background ratio (TBR) was observed at 48 hours post‐injection, identifying this as the optimal time for NIR‐II fluorescence‐guided tumor resection. To assess the nanoprobe's capacity for intraoperative detection of small tumors, a multiple microtumor model was established. The intraoperative fluorescence signals closely correlated with bioluminescence imaging results (**Figure** **8**A), demonstrating the accuracy of NIR‐II fluorescence imaging in delineating tumor margins. Corresponding HE staining confirmed that the diameters of seven microtumors excised under NIR‐II guidance ranged from approximately 0.5 to 3 mm, with clear differentiation of tumor boundaries from adjacent normal tissue (Figure 8B; Figure S36, Supporting Information). Importantly, microtumors T3, T4, and T5, each under 1 mm in diameter, were accurately identified, highlighting the effectiveness of RVLu@ICG fluorescence imaging for detecting small residual lesions during surgery.

### 2.10 NIR‐II Fluorescence‐Guided Real‐Time Tumor Resection in Intramuscular Tumor‐Invasion Model

An intramuscular tumor‐invasion model was constructed to evaluate the feasibility of our RVLu@ICG‐based NIR‐II nanoprobes for precisely identifying the tumors with a fluorescence imaging system. First, CT26‐Luc cells were implanted intramuscularly into BALB/c mice until tumor volume reached approximately 300–400 mm^3^, which was confirmed by the bioluminescence imaging (Figures S37–S39, Supporting Information). Then, the tumor‐bearing mice were anesthetized by isoflurane under aseptic conditions. As shown in Figure 8C, 48 hours after RVLu@ICG administration, tumor margins were distinctly visualized via the NIR‐II fluorescence imaging system (Figure 8C I,IV). Initial excision of the primary tumor (P1) was performed under white light, followed by assessment of the surgical margins using NIR‐II imaging (Figure 8C II,V). Residual NIR‐II fluorescence detected in the surgical bed prompted additional resection (P2) in certain cases. The resection area was subsequently re‐examined under 808 nm laser excitation to verify the complete absence of fluorescence signals, confirming thorough tumor clearance (Figure 8C III,VI). Peritumoral normal tissue was collected as a standard control. *Ex vivo* NIR‐II fluorescence imaging revealed that both P1 and P2 exhibited strong fluorescence signals, whereas the muscle tissue remained dark (Figure 8E). These findings were consistent with intraoperative fluorescence imaging results (Figure 8C IV,V). HE staining confirmed that the resected tissues exhibiting fluorescence signals were tumor tissues, while non‐fluorescent tissues were identified as normal tissue (Figure 8F). This unequivocally demonstrated that RVLu@ICG nanoprobes effectively differentiate tumors from normal tissues, assisting surgeons in achieving negative surgical margins. In contrast, in the ICG group, most residual tumors lacked fluorescence signals, underscoring the tumor‐targeting and retention limitations of ICG, which is commonly used in surgical oncology (Figure 8D).

To evaluate tumor recurrence and metastatic spread, bioluminescence imaging was performed 14 days post‐surgery. Remarkably, no local tumor relapse or distant metastasis was observed in the RVLu@ICG group following NIR‐II fluorescence‐guided resection (Figure S39, Supporting Information). Conversely, 80% of mice in the ICG‐treated group and all mice in the control cohort exhibited tumor regrowth at the original site (Figures S38 and S37, Supporting Information). These results underscore the superior efficacy of RVLu@ICG nanoprobes in achieving thorough tumor removal. Animals were monitored bi‐daily for survival assessment until tumor volumes reached 2000 mm^3^ or death occurred. Kaplan–Meier analysis (Figure 8G) revealed a significant survival advantage in the RVLu@ICG‐guided surgery group relative to ICG‐ and white light‐guided groups.

### 2.11 NIR‐II Fluorescence‐Guided Real‐Time Tumor Resection in Peritoneal Metastasis Model

We further explored the potential of RVLu@ICG‐based NIR‐II nanoprobes for intraoperative imaging in a CRC peritoneal metastases model. Initially, bioluminescence imaging confirmed tumor presence and provided an approximate localization (Figure S42, Supporting Information). Under sterile conditions, mice were anesthetized with isoflurane, and tumor excision was performed sequentially guided by NIR‐II fluorescence imaging (Figure 8H; Figure S40, Supporting Information). The tumor boundaries (1–8) were clearly visualized using NIR‐II imaging (Figure 8H; Figures S40 and S41, Supporting Information). HE staining of resected tissues verified the accurate delineation of tumor margins (Figure 8I). To assess potential residual recurrence, bioluminescence imaging was performed on day 14 post‐surgery, revealing no signs of local recurrence or metastasis (Figure S42, Supporting Information).

## 3 Discussion

Neoadjuvant radiotherapy has established itself as a cornerstone in the multidisciplinary management of patients with locally advanced colorectal cancer. Although conventional RT has demonstrated certain benefits in improving local tumor control and patient survival, its therapeutic efficacy remains limited by several factors, including treatment‐related toxicities and intrinsic radioresistance of tumor cells. A growing body of evidence suggests that RT induces ICD, promoting the release of TAAs and DAMPs, which play a critical role in initiating antitumor immune responses. Nevertheless, CRC frequently exhibit an immunologically “cold” TME, characterized by scarce infiltration of immune effector cells and subdued adaptive immune activation,^[^ ^41^ ^]^ thereby limiting the immunostimulatory effects of RT. Additionally, tumor cell radioresistance further compromises RT's capacity to induce potent immune responses. Consequently, overcoming these challenges through radiosensitization strategies that can remodel the tumor immune microenvironment, enhance tumor immunogenicity, and potentiate antitumor immunity has become a key scientific objective in improving the therapeutic outcomes of RT in CRC.

Over the last decade, a variety of nanomaterials have been engineered and evaluated for their potential to enhance radiosensitivity, with high atomic number (Z) lanthanide nanoparticles gaining significant research interest. These materials have shown promise due to their ability to enhance radiation therapy by increasing the production of ROS, improving tumor targeting, and amplifying the overall therapeutic effect. Radiosensitization effects were observed in high Z lanthanide nanoparticles constructed of Neodymium (Nd) and Gadolinium (Gd).^[^ ^42^, ^43^ ^]^ Unfortunately, the RT sensitization effect of Nd and Gd is not ideal and still requires a relatively high radiation dose due to their lower atomic numbers. In particular, Lu has a higher atomic number than Gd, exhibiting a better RT sensitization effect. Compared with reported Gd‐based radiation sensitizers like R&HV‐Gd@ICG,^[^ ^34^ ^]^ NPs‐Bev^[^ ^44^ ^]^ or VGd@ICG‐FA,^[^ ^29^ ^]^ Our RVLu@ICG probe demonstrates superior radiosensitization efficacy, significantly enhanced local tumor control, and potential prognostic improvement capabilities compared to conventional modalities.

Furthermore, integrin α_v_β_3_ is abundantly expressed on neovascular endothelial cells as well as CRC cells^[^ ^45^, ^46^ ^]^ making it a widely recognized tumor biomarker.^[^ ^47^, ^48^ ^]^ And c(RGDfK) can specifically bind to integrin α_v_β_3_. In this study, NIR‐II nanoparticles RVLu@ICG was synthesized by integrating ICG into a c(RGDfK) modified biodegradable hollow virus‐like Lu_2_O_3_ nanoprobe. In addition to passive accumulation mediated by the enhanced permeability and retention (EPR) effect,^[^ ^49^ ^]^ conjugating with specific tumor‐targeting elements c(RGDfK) could specificity and excellently enable RVLu@ICG to target cancer cells in vitro and identify tumor in vivo actively. ICG are useful for NIR‐II imaging, facilitating the tracking of NPs accumulation within tumors and more precision tumor RT. Blood analysis and histological staining of major organs jointly supported the reliable biocompatibility of RVLu@ICG, making them candidates for anti‐tumor therapy. Following the evaluation of the exceptional performance of RVLu@ICG, our in vitro experiments revealed their remarkable capacity to enhance RT efficacy and further activated RT‐related ICD response by increasing ROS production, intensifying oxidative stress, and destroying the integrity of the nucleus which collectively improve the therapeutic response to radiation.^[^ ^50^ ^]^ In vivo studies also suggested that the RVLu@ICG + RT effectively enhanced tumor cells apoptosis and further activated ICD response induced by RT, which could promote more secretion of cytokine and recruitment and infiltration of CTLs. Following this, DCs and macrophages migrated to phagocytose the necrotic tumor cells, facilitating the presentation of tumor‐derived antigenic peptides to T cells. This process activated tumor‐specific T cell responses, resulting in the elimination of subcutaneous tumors and the promotion of immunogenic cell death, thereby eliciting a potent antitumor immune reaction.

In addition to preoperative neoadjuvant therapy, precise intraoperative resection of tumor lesions plays a critical role in determining the prognosis of CRC patients. Although ICG has been utilized for NIR‐II imaging‐guided surgery,^[^ ^51^ ^]^ its clinical application remains challenging due to severe photobleaching. The encapsulation of ICG within inorganic nanoparticles significantly enhances its photostability. In both in vitro and in vivo experiments presented herein, thanks to the accurate tumor targeting ability, RVLu@ICG exhibited superior NIR‐II imaging performance, characterized by strong fluorescence emission beyond 1000 nm. Remarkably, free ICG fluorescence rapidly diminished within 40 minutes of continuous laser irradiation, whereas RVLu@ICG maintained nearly 100% of its fluorescence intensity, demonstrating outstanding photostability and highlighting its promising potential for applications in surgical navigation. By comparing to the VLu@ICG, RVLu@ICG achieved a high TBR of ≈6.5 for in vivo tumor imaging sufficiently to discriminate tumor tissue intraoperatively. More importantly, the tumor tissue retained a significant fluorescent signal (TBR = 4.5) even 72 hours post‐injection, demonstrating the prolonged imaging window of RVLu@ICG. The 24–72 hour time frame offers considerable flexibility for scheduling probe injections and planning surgical procedures, making it advantageous for clinical applications where precise timing is crucial. The nanoprobe further demonstrated the capability to detect microtumors as small as 1 mm^3^ in volume within microtumor models and effectively guided accurate and complete tumor resection in both intramuscular invasion and peritoneal metastasis models under NIR‐II fluorescence imaging, facilitated by the c(RGDfK) modification. Collectively, these results establish RVLu@ICG as a promising tool for precise surgical intervention with strong potential for clinical translation.

Despite the favorable therapeutic efficacy and fluorescence imaging capabilities of the developed Lu‐based nanoprobes, their fabrication process remains intricate, involving sequential steps such as VLu synthesis, ICG incorporation, and c(RGDfK) functionalization. Undoubtedly, the purification required at each stage is labor‐intensive, making comprehensive optimization of all components challenging. Moreover, comprehensive studies are still required to elucidate the long‐term biosafety, metabolic implications, and in vivo fate of the nanoprobes. Future investigations involving large primate models are essential to assess systemic distribution and targeted delivery to colorectal tumors.

In conclusion, we engineered an innovative multifunctional NIR‐II emitting degradable fluorescent nanoprobe RVLu@ICG with excellent biocompatibility and high photostability, for the RT sensitization, immune microenvironment remodeling and accurate navigation of surgery. Owing to the pronounced X‐ray absorption efficiency of the high atomic number element Lu to generate ROS, RVLu@ICG enhanced the radio‐sensitization of CRC tumors while further remodeling the tumor immune microenvironment through the radiosensitizing effect, thereby stimulating immunity to amplify immune killing effects. In addition, the probe could be used for accurate tumor removal under the guidance of NIR‐II fluorescence imaging leading to radical resection and improve overall survival in various tumor‐bearing models. More encouragingly, this nanoprobe is non‐toxic and does not cause organ damage or acute harm, highlighting its promising potential for future clinical translation. This multifunctional probe enables a comprehensive precision treatment strategy for CRC. In the preoperative phase, it facilitates maximal tumor cell eradication and tumor volume reduction through neoadjuvant RT combined with immune microenvironment remodeling. Subsequently, it allows for the precise intraoperative resection of residual microscopic lesions under NIR‐II fluorescence guidance. This integrated approach holds significant promise for improving patient survival and prognosis, and may serve as a viable candidate for clinical application in cancer therapy.

## 4 Experimental Section

### Preparation of RVLu@ICG

RVLu&NPs (10 mg) were immersed in 5 mL ICG solution (2 mg mL^−1^) for dark‐stirring over 4 h, then subjected to centrifugation (10,000 rpm) and triple aqueous rinsing.

### ICG and RVLu@ICG Photostabilities

RVLu@ICG and ICG were suspended in PBS, and their NIR‐II fluorescence images were captured under continuous 808 nm laser irradiation at various time point. RVLu@ICG and ICG were dissolved in PBS and stored at room temperature under dark conditions for different periods. Then their NIR‐II fluorescence images were measured at 808 nm. RVLu@ICG was dispersed to aqueous solutions with pH 7.4, 6.5, 5.5 for different times. Then centrifuge to collect the supernatant, and measure the fluorescence intensity at 808 nm.

### Cell Culture

CT26 murine colon carcinoma cells were procured from the American Type Culture Collection (ATCC, USA), with CT26‐Luc cells acquired from Shanghai Zhong Qiao Xin Zhou Biotechnology. Both lineages were propagated in RPMI‐1640 containing 10% FBS and 1% penicillin‐streptomycin, incubated at 37 °C under 5% CO_2_ humidified atmosphere. Based on the morphological characteristics and growth behavior of the cells, it was confirmed that they were not contaminated.

### Radiosensitization of RVLu@ICG in Vitro

Intracellular reactive oxygen species production was assessed using a DCFH‐DA fluorescence assay. Cell slides were prepared in accordance with the protocol described above. Samples treated with RVLu@ICG were incubated with a final concentration of 20 µM to evaluate ROS levels. The RT group received 4 Gy X‐ray irradiation. ROS fluorescence signals were observed using a Nikon AI‐MP microscope under the FITC channel.

### Immunogenic Cell Death‐Related Assays in Vitro

CT26 cells were cultured at a density of 5 × 10^5^ cells mL^−1^ for 24 hours and then treated with either PBS or RVLu@ICG for an additional 24 hours. Following triple PBS rinses, the cells underwent X‐ray exposure as previously described. The samples were then fixed in 4% paraformaldehyde and permeabilized with Triton X‐100. Following blocking with bovine serum albumin (BSA), they were incubated overnight at 4 °C with primary antibodies against HMGB1 and CRT. Post‐incubation, cells were washed 3 times, followed by a 1‐hour staining step and a 15‐minute incubation with DAPI and goat anti‐rabbit IgG. Immunofluorescence imaging was finally performed using CLSM.

### RNA Sequencing Analysis

When CT26 tumors attained a volume near 100 mm^3^, mice were randomly assigned to two groups (n = 3) and administered either PBS or a combination of RVLu@ICG + RT. Forty‐eight hours following treatment, tumors were excised, rapidly frozen in liquid nitrogen, and submitted to Shanghai OE Biotech Co., Ltd. for transcriptomic profiling via RNA sequencing.

### RT of RVLu@ICG Nanoprobe in Vivo

To establish the xenograft model, tumor‐bearing mice with 50 ± 5 mm^3^ lesions were randomized into four cohorts (n = 5): (I) PBS, (II) RVLu@ICG, (III) RT, (IV) RVLu@ICG+RT. Cohorts II and IV received tail vein administration of 100 µL RVLu@ICG suspension (2 mg/mL), while cohorts III and IV underwent fractional X‐ray irradiation (8 Gy, 6 MeV, Varian Medical Systems). Physiological parameters (body mass) and neoplastic progression (tumor dimensions) were monitored every 48 hours. And the tumor growth inhibition (TGI) was calculated using the formula: TGI = (1 – RTV_C_/RTV_E_) × 100%, where RTV_C_ was the relative tumor volume of the control group. RTV_E_ was the relative rumor volume of the experimental group. Terminal biospecimens were excised for macroscopic documentation and gravimetric analysis to quantitatively evaluate therapeutic outcomes.

### Immune Response Evaluation in Vivo

To assess in vivo immune activation, CT26 tumor‐bearing mice were randomly allocated into four groups (n = 5 each) receiving PBS, RVLu@ICG, RT, or the combination of RVLu@ICG + RT. Forty‐eight hours post‐treatment, tumors and corresponding draining lymph nodes were harvested to prepare single‐cell suspensions. For analysis of DCs maturation, lymph node‐derived cells were stained with APC‐conjugated anti‐CD11c, PE‐labeled anti‐CD86, and FITC‐tagged anti‐CD80 antibodies, followed by flow cytometric evaluation. To assess cytotoxic T lymphocytes (CTLs) in tumor tissues and spleens, samples were stained with FITC anti‐CD45, Brilliant Violet 510™ anti‐CD3, Brilliant Violet 421™ anti‐CD4, and PE/Cyanine7 anti‐CD8a, then subjected to quantitative flow cytometry. Additionally, portions of tumor tissues were processed for immunofluorescence staining targeting CD8 and CD86. Serum samples were also collected to quantify cytokine levels via ELISA.

### Microtumor Identification and Resection under NIR‐II Fluorescence‐Guided

Mice bearing multiple microtumors (n = 4) were imaged using the IVIS‐II bioluminescence system (PerkinElmer, Waltham, MA, USA) to precisely identify tumor locations. Following localization, RVLu@ICG was administered intravenously, and after 48 hours, the animals were euthanized for subsequent analysis. NIR‐II fluorescence imaging was utilized to facilitate surgical excision of tumors. Resected specimens, including adjacent non‐tumorous tissues, were then subjected to *ex vivo* NIR‐II imaging and further examined through histopathological evaluation.

### NIR‐II Fluorescence‐Guided Real‐Time Tumor Resection in Intramuscular Tumor‐Invasion Model

CT26 Luc tumor cells were implanted near the right leg of mice, enabling gradual tumor infiltration into the muscle. Tumor presence and localization were verified through bioluminescence imaging. Surgical resection, conducted under white light to emulate clinical practice, involved both visual inspection and palpation of the tumor. In the RVLu@ICG and ICG treatment cohorts, NIR‐II fluorescence imaging was utilized intraoperatively to detect residual signal within the surgical field. Additional tissue removal was carried out until fluorescence was no longer observed. Surrounding muscle tissue was also excised for comparative purposes. All collected samples were then subjected to NIR‐II imaging, followed by histopathological evaluation. In contrast, animals in the control group underwent complete tumor resection guided solely by standard white‐light visualization. 14 days post‐surgery, bioluminescent imaging was conducted on all mice to monitor tumor recurrence.

### NIR‐II Fluorescence‐Guided Real‐Time Tumor Resection in Peritoneal Metastasis Model

Approximately 5×10^5^ CT26 Luc cells per dish were initially cultured in flasks containing 8 mL of DMEM supplemented with 1% antibiotics and 10% FBS, and maintained in a CO₂ atmosphere at 37 °C for 1 day. After incubation, the cells were collected by centrifugation, resuspended in sterile PBS, and then administered intraperitoneally into female mice at a dosage of 5×10^7^ cells one mouse. Bioluminescence imaging was used to confirm tumor presence and estimate its location. Surgical resection was performed 7 days post‐injection, following the same procedure as for intramuscular tumor models.

## Conflict of Interest

The authors declare no conflict of interest.

## Supporting information



Supporting Information

## Data Availability

The data that support the findings of this study are available from the corresponding author upon reasonable request.
