# Supplementary material for: Lutetium‐Based Nanoprobes for Radiosensitization with Immune Microenvironment Remodeling and NIR‐II Fluorescence Imaging‐Guided Surgery in Colorectal Cancer
Source: Adv Sci (Weinh). 2025 Jul 21;12(38):e10136. doi: 10.1002/advs.202510136 (PMC12520561; doi:10.1002/advs.202510136)
Supplement: Supplementary file 1 — Supporting Information [file ADVS-12-e10136-s001.docx]

**Lutetium-Based Nanoprobes for Radiosensitization with Immune Microenvironment Remodeling and NIR-II Fluorescence Imaging-Guided Surgery in Colorectal Cancer**

*Yongying Dang^#^, Xianzhi Liu^#^, Zifan Zheng^#^, Ao Wang, Ying Huang, Zhong Luo, Haina Tian, Siyaqi Li, Qiang Luo^*^, Peiyuan Wang^*^, and Weiling He^*^*

Y. Dang, X. Liu, Z. Zheng, Z. Luo, W. He

Department of Gastrointestinal Surgery

Xiang'an Hospital of Xiamen University

School of Medicine

Xiamen University

Xiamen 361100, P. R. China

Email: wlhe@xah.xmu.edu.cn

A. Wang, S. Li, Q. Luo, P. Wang

Key Laboratory of Design and Assembly of Functional Nanostructures

Fujian Institute of Research on the Structure of Matter

Chinese Academy of Sciences

Fuzhou 350002, P. R. China

Email: [luoq@fjirsm.ac.cn](mailto:luoq@fjirsm.ac.cn); [wangpeiyuan@fjirsm.ac.cn](mailto:wangpeiyuan@fjirsm.ac.cn)

Y. Huang

Department of Etiology and Carcinogenesis

National Cancer Center/National Clinical Research Center for Cancer/Cancer Hospital

Chinese Academy of Medical Sciences and Peking Union Medical College

Beijing, 100021, P. R. China

H. Tian

School of Chemistry and Materials Engineering & Engineering Research Center of Biomass Conversion and Pollution Prevention Control of Anhui Provincial Department of Education

Fuyang Normal University

Fuyang 236037, P.R. China

Keywords: Colorectal cancer; Radiotherapy sensitization; Immune microenvironment remodeling; NIR-II fluorescence imaging; Precise discrimination of surgical margins

**Supplementary materials and methods**

*Materials:* NaOH, ammonia aqueous, Lu(NO_3_)_3_·6H_2_O, ethanol, cyclohexane, dimethyl sulfoxide (DMSO), and acetone were obtained from Sinopharm Chemical Reagent Co., Ltd. Tetraethyl orthosilicate (TEOS), hexadecyltrimethylammonium bromide (CTAB), and (3-Aminopropyl) triethoxysilane (APTES) were purchased from Sigma-Aldrich. Indocyanine green (ICG) was purchased from BBI life science Co., Ltd, and c(RGDfK) was supplied by GL Biochem (Shanghai) Ltd. Fetal bovine serum (FBS), trypsin-EDTA (0.25%), and RPMI 1640 medium were purchased from Gibco (USA). BCA kit, DAPI, and Annexin V-FITC/PI Cell Apoptosis Kit were brought from Shanghai Beyotime Biotechnology Co., Ltd. Anti-IgG, anti-Ki67, anti-Tunel, anti-CRT, and anti-HMGB1 were ordered from Abcam Trading Co., Ltd. (Shanghai, China). The FITC anti-mouse CD45, Brilliant Violet 510™ anti-mouse CD3, Brilliant Violet 421™ anti-mouse CD4, PE/Cyanine7 anti-mouse CD8a, APC anti-mouse CD11c, PE anti-mouse CD86, and FITC anti-mouse CD80 were purchased from Biolegend (USA). All chemicals were used as received without further purification. The deionized (DI) water was purified and prepared by Milli-Q water (18 MΩ/cm) for the whole experiment.

*Preparation of VLu*: Initially, approximately 750 mg of cetyltrimethylammonium bromide (CTAB) was dissolved in 60 mL of deionized water. Subsequently, 0.75 mL of 0.1 M NaOH was added, and the mixture was stirred at 60°C for 30 minutes. Next, 16 mL of cyclohexane along with 4 mL of tetraethyl orthosilicate (TEOS) was introduced, and the reaction proceeded at 60°C for 72 hours. After removing the upper phase, the precipitate was centrifuged and washed three times with deionized water to yield virus-like mesoporous silica nanoparticles (VSi). Then, 100 mg of VSi, 280 mg of Lu(NO_3_)_3_·6H_2_O, and 50 mL of deionized water were combined and heated at 90°C for 30 minutes. Following this, 105 mg of hexamethylenetetramine was added, and the solution was stirred at 90°C for 4 hours to form virus-like lutetium nanoparticles. After a water wash, the silica template was etched away using 0.5 M Na_2_CO_3_ at 80°C for 12 hours, and three further washes with deionized water produced the final hollow virus-like lutetium nanoparticles (VLu).

*Preparation of RVLu*: Initially, 100 mg of synthesized VLu and 0.75 mL of APTES were dissolved in 30 mL of anhydrous ethanol and stirred for 12 hours at 75 °C. The mixture was then washed three times with deionized water to yield amino-functionalized nanoparticles (VLu &NPs-NH_2_). Subsequently, these particles were covalently linked to c(RGDfK) peptides via an EDC/NHS reaction conducted at 4 °C for 12 hours. After another series of three water washes, the final RVLu&NPs were re-dispersed in water for subsequent experiments.

*Characterizations:* TEM images were captured with a Hitachi HT-7800 microscope (Hitachi Ltd, Tokyo, Japan) operating at 200 KV, while SEM (FEI, Hillsboro, Oregon, USA) was employed to assess nanoparticle morphology. Elemental mapping was performed on a Talos F200s microscope (Thermo Fisher Scientific, Waltham, Massachusetts, USA), and HRTEM images were acquired using another Thermo Fisher Scientific instrument. Fluorescence spectra were recorded with an FLS980 spectrometer (Edinburgh Instruments Ltd, Livingston, UK), and UV-vis-NIR spectra were collected using a Cary 5000 spectrophotometer (Agilent Technologies, Santa Clara, California, USA). Finally, the zeta potentials were determined using a Malvern Zetasizer (Malvern, UK).

*Nanoparticle Degradation*: RVLu@ICG was dispersed in aqueous buffers (pH 7.4, 6.5, 5.5) under distinct temporal conditions. Both parent solutions and degradation products underwent TEM characterization at predetermined intervals.

*Cytotoxicity Assay*: CT26 cell cytotoxicity was assessed via CCK-8 assay. Cells were plated in 96-well plates (6×10^3^ cells/well) and incubated for 24 h. Medium was replaced with fresh medium containing RVLu@ICG at varying concentrations (0-80 μM). Post 24-hour treatment, viability was calculated using: Viability (%) = [(At - Ab)/ (Ac - Ab)] × 100%, where At = absorbance of test groups, Ac = untreated controls, and Ab = blank wells.

*Cellular Uptake*: To assess the impact of virus-like morphology on cellular uptake, 1 × 10^5^ CT26 cells were seeded in 12-well slide chambers per well. Incubated for 24 hours in a 5% CO_2_/air environment at 37°C. Subsequently, virus-like mesoporous Lu_2_O_3_ nanoparticles loaded with ICG (VLu@ICG) and standard mesoporous Lu_2_O_3_ nanoparticles (MLu@ICG) were added at an identical ICG concentration (4 μg/mL). Following a 4-hour incubation, cells were rinsed with PBS and fixed with paraformaldehyde. Subsequently, after DAPI nuclear staining, CLSM images were acquired using a Leica DM2700 Portho fluorescence microscope.

To determine the effect of c(RGDfK) on uptake, CT26 cells were similarly cultured in 12-well slide chambers for 24 hours, after which RPMI-1640 medium containing RVLu@ICG, VLu@ICG, or ICG (at equivalent concentrations) was introduced and incubated for various durations (15 min, 30 min, 1 h, 2 h, and 4 h). After each incubation, the cells were washed with PBS and Flow cytometric analysis was performed using a CytoFlexS system, with untreated cells serving as controls. In a separate experiment, cells were pretreated with c(RGDfK) (40 μg/mL) for 1.5 hours before the addition of RVLu@ICG, followed by an additional 4-hour incubation, and the MFI was subsequently measured.

For a more direct visualization of uptake differences, CT26 cells were seeded in 12-well slide chambers and cultured for 24 hours. One group was directly co-cultured with RVLu@ICG, VLu@ICG, or ICG for 4 hours, while another group was pretreated with 40 μg/mL c(RGDfK) for 1.5 hours prior to a 4-hour incubation with RVLu@ICG. After treatment, the cells were washed with PBS, fixed with paraformaldehyde, stained with DAPI, and then imaged by CLSM.

*Radiosensitization of RVLu@ICG in Vitro*: CT26 cells were plated in 6-well plates at a density of 1 × 10^3^ cells per well and exposed to RVLu@ICG at concentrations of 0 or 20 μM for 24 hours. Following treatment, cells received X-ray irradiation at doses of 0 or 4 Gy. After rinsing twice with PBS, they were cultured in fresh DMEM for an additional 8 days. Colonies were then fixed using methanol and stained with crystal violet. The surviving fraction was determined by dividing the number of colonies formed by the product of the initial seeding number and the plating efficiency. Data are expressed relative to the untreated control group.

Apoptotic progression was quantitatively evaluated through flow cytometric analysis employing a dual-staining protocol with Annexin V-FITC and propidium iodide. Cellular specimens were plated in 6-well culture dishes at an optimized density of 1×10^6^ cells/well and subjected to therapeutic interventions including RVNd@ICG nanoparticles, RVGd@ICG nanoparticles, RVLu@ICG complexes, or radiation therapy (RT). Following 24-hour incubation under standard culture conditions, membrane phospholipid redistribution and cytoplasmic integrity were assessed through fluorescent ligand binding. Quantitative data acquisition was performed using a flow cytometer, applying quadrant gating strategies to differentiate viable, early apoptotic, late apoptotic, and necrotic populations.

*RVLu@ICG Biosafety in Vivo*: Mice were sourced from Gempharmatech Co., Ltd. (Jiangsu, China) and housed at the Xiamen University Laboratory Animal Center. All procedures received approval from the Institutional Animal Care and Use Committee of Xiamen University and adhered strictly to established guidelines (Ethics Approval: No. XMULAC20240074). To evaluate the *in vivo* toxicity of RVLu@ICG, female BALB/c mice (7 weeks) were allocated into different groups randomly (n = 3). Two groups were intravenously administered 100 μL of RVLu@ICG (2 mg/mL) or an equivalent volume of PBS. Mouse body weights were recorded every two days, and on day 28 post-injection, blood samples were obtained for routine hematological and biochemical analyses. Following euthanasia, major organs—including the liver, brain, spleen, kidney, lung, and heart—were collected for histopathological examination. In parallel, two additional groups received RVLu@ICG via intravenous injection, with blood and organ samples collected on days 1 and 7 for the same assessments.

*Targeted Imaging, Biodistribution, and Block Imaging in Vivo*: Female BALB/c mice were employed in this study. Each animal was subcutaneously injected in the right hind limb with 45 μL of PBS containing 1 × 10^4^ CT26-Luc cells. Tumor size was calculated using the ellipsoid volume formula: length × width^2^ × 0.5. Upon reaching a volume of approximately 200-300 mm^3^, the mice were randomly assigned to three groups (n = 3) and treated with RVLu@ICG, VLu@ICG, or free ICG at a dose of 1 mg/kg. Fluorescence imaging was performed at various time intervals using a NIR-II imaging system.

For biodistribution studies, tumor-bearing mice received RVLu@ICG, and at 48 hours post-administration, organs including the tumor, heart, liver, kidney, brain, lung, spleen, and muscle were excised for NIR-II fluorescence analysis. In receptor-blocking experiments, c(RGDfK) was administered intravenously at 40 mg/kg 30 minutes prior to imaging to evaluate the influence of integrin inhibition on tumor targeting efficiency.

*Histochemical Analysis*: CT26 tumor-bearing mice with approximately 50 mm^3^ tumor volumes were randomly allocated into four cohorts (5 mice per group). Intravenous injections of PBS or 100 μL RVLu@ICG (2 mg/mL concentration) were administered. X-ray irradiation (8 Gy) was delivered 48 hours post-administration. Three animals per group underwent euthanasia 18 days post-treatment for tumor excision. Resected tissues underwent paraffin embedding, followed by 4 μm sectioning and sequential staining with HE solution, Ki67 (Abcam ab15580, 1:100 dilution), TUNEL assay reagents, Caspase-3 antibody (Abcam ab179475, 1:500 dilution), and Bax (Abcam ab32503, 1:250 dilution). Histochemical analysis was conducted using a Leica DM2700 P microscope (USA), with subsequent quantification performed through ImageJ software.

*Statistical Analysis:* All experiments were independently performed a minimum of three times. Statistical evaluation of group differences was conducted using one-way or two-way ANOVA, followed by Tukey’s post hoc test for multiple comparisons, utilizing GraphPad Prism 8.0 software. Data are presented as mean ± standard deviation (SD). Survival outcomes were analyzed using the Kaplan–Meier method. A p-value of less than 0.05 was considered indicative of statistical significance (**P* < .05, ***P* < .01, ****P* < .001, *****P* < .0001).

**Supplementary Figures**


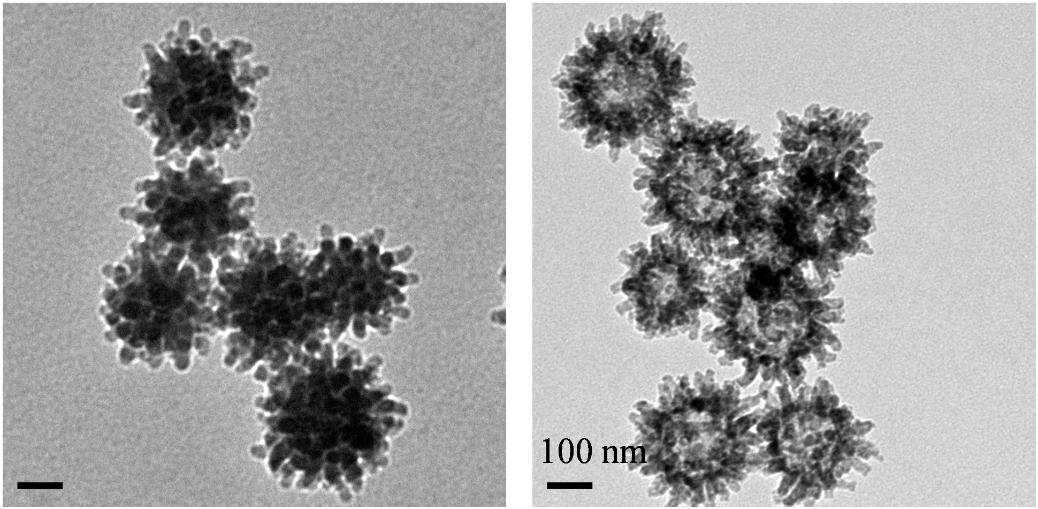
**Figure S1.** TEM images of six magnified VSi (left) and nine magnified VLu (right). TEM: transmission electron microscopy. The scale bar represents 100 nm.


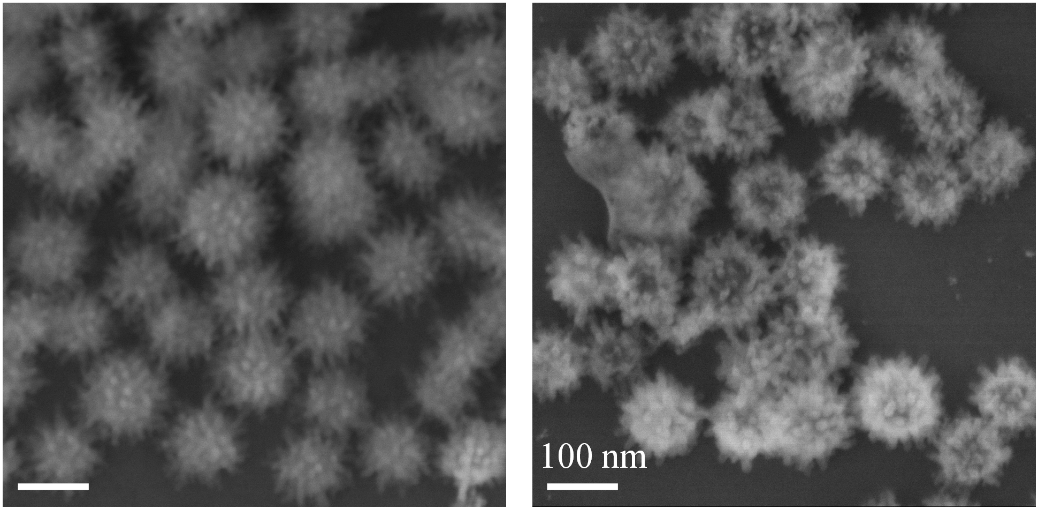


**Figure S2.** SEM images of VSi (left) and VLu (right) in magnification. SEM: scanning electron microscopy. The scale bar represents 100 nm.


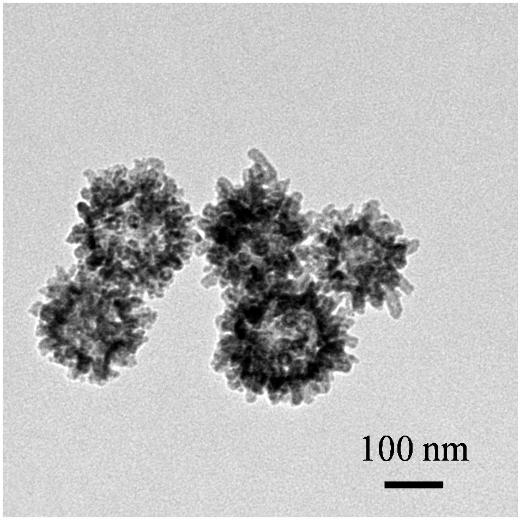
**Figure S3.** TEM image of RVLu. TEM: transmission electron microscopy. The scale bar represents 100 nm.


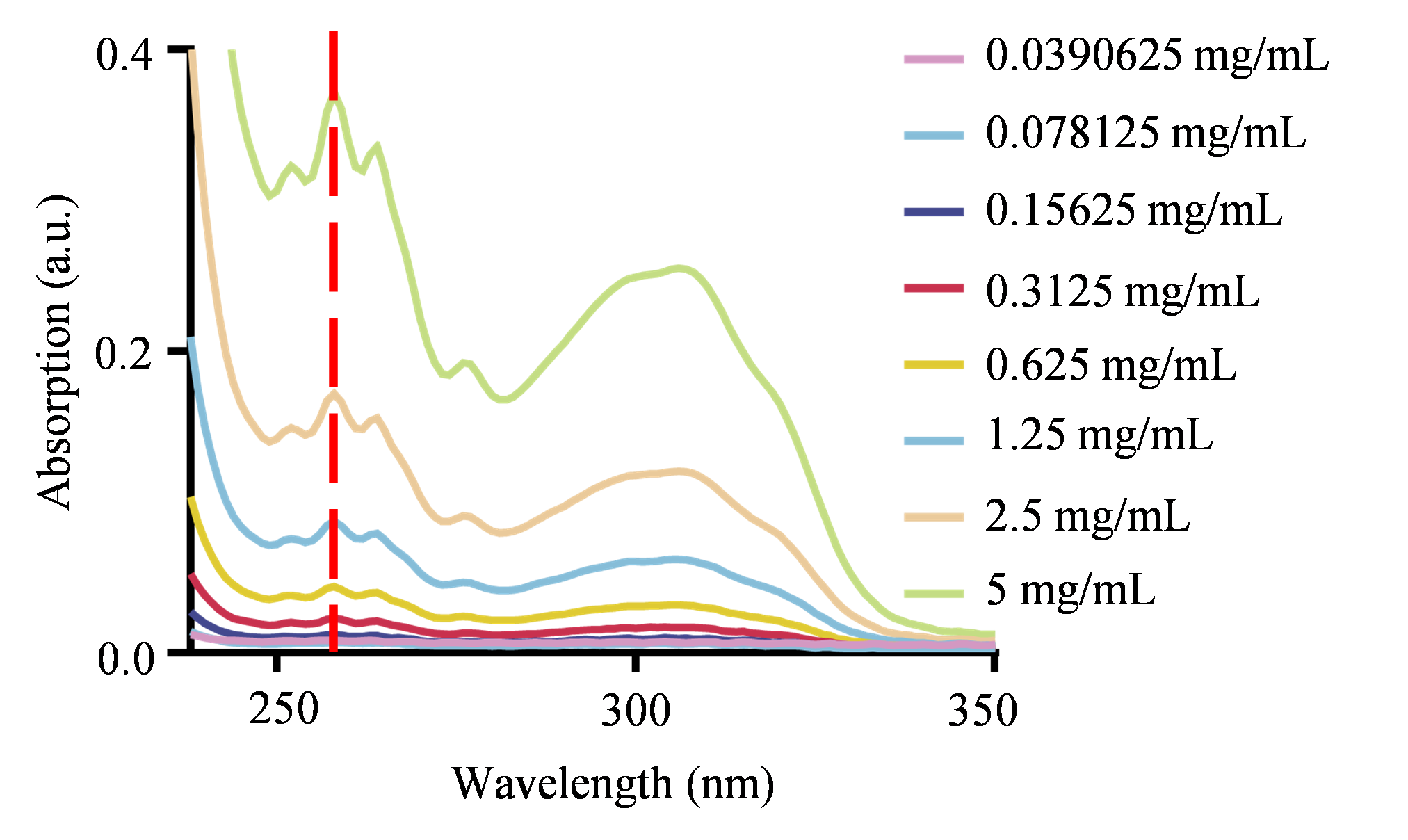
**Figure S4.** UV-vis spectra of c(RGDfK) with different concentrations.


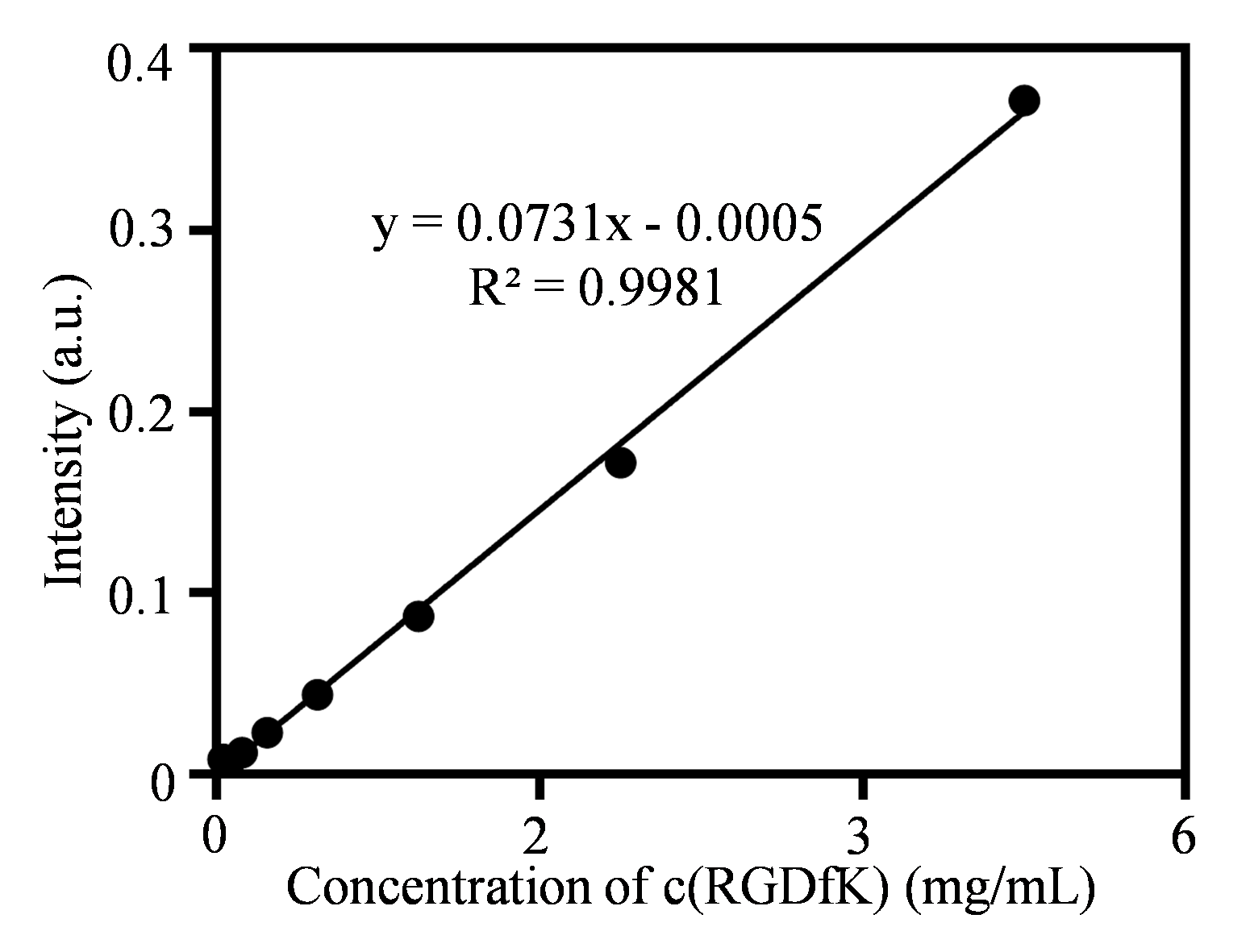
**Figure S5.** The standard curve of UV absorption (at 258 nm) towards c(RGDfK) concentration.


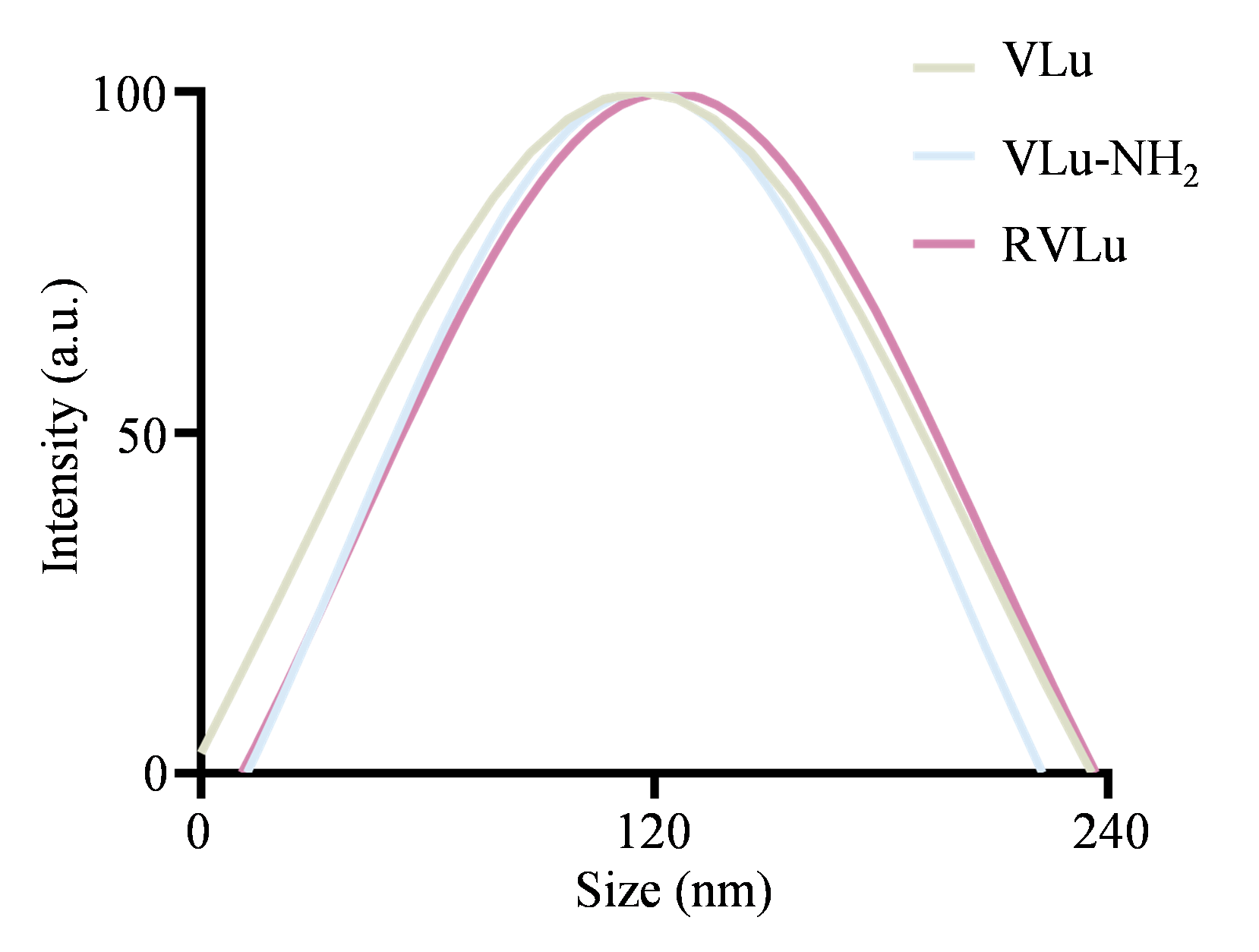


**Figure S6.** The size distribution of VLu, VLu-NH_2_ and RVLu.


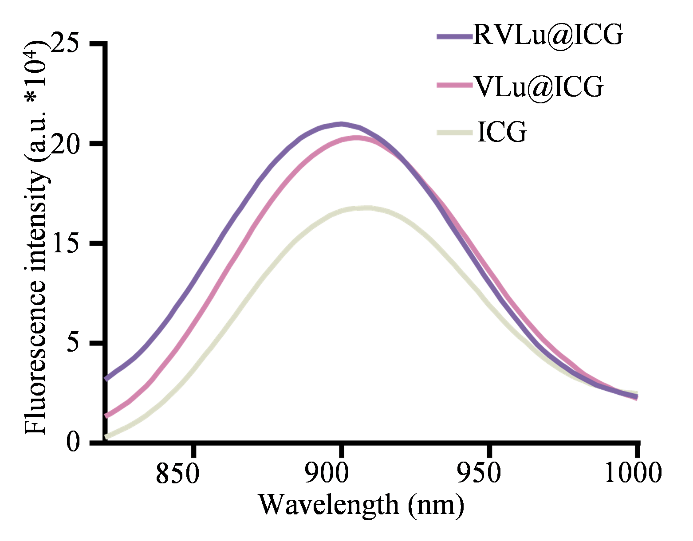
**Figure S7.** Fluorescent spectra of ICG, VLu@ICG, and RVLu@ICG under 808 nm laser excitation in NIR-I.


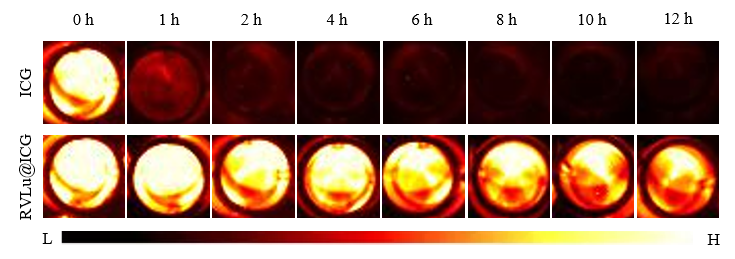


**Figure S8.** NIR-II fluorescence images of ICG (up) and RVLu@ICG (down) dispersed in PBS for different time at room temperature, irradiated by 808 nm laser.


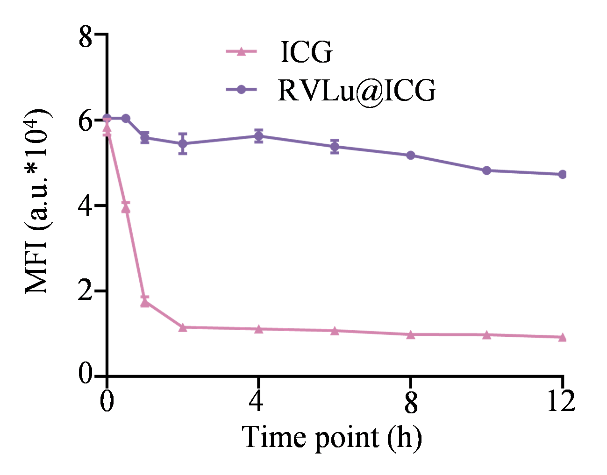
 **Figure S9.** The corresponding fluorescence intensity in **Figure S8**.


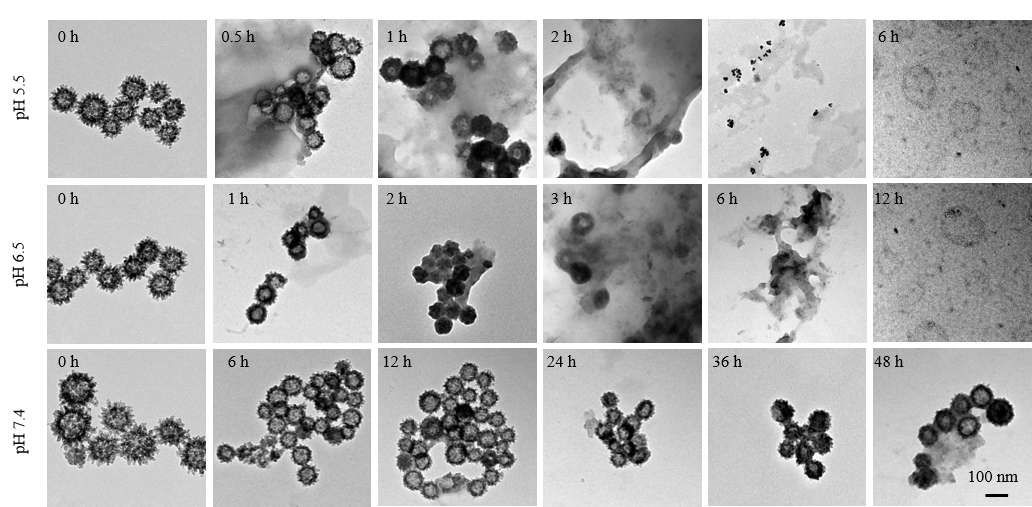


**Figure S10.** TEM images of RVLu after incubation in buffers with different pH values (7.4, 6.5 and 5.5) for various periods.


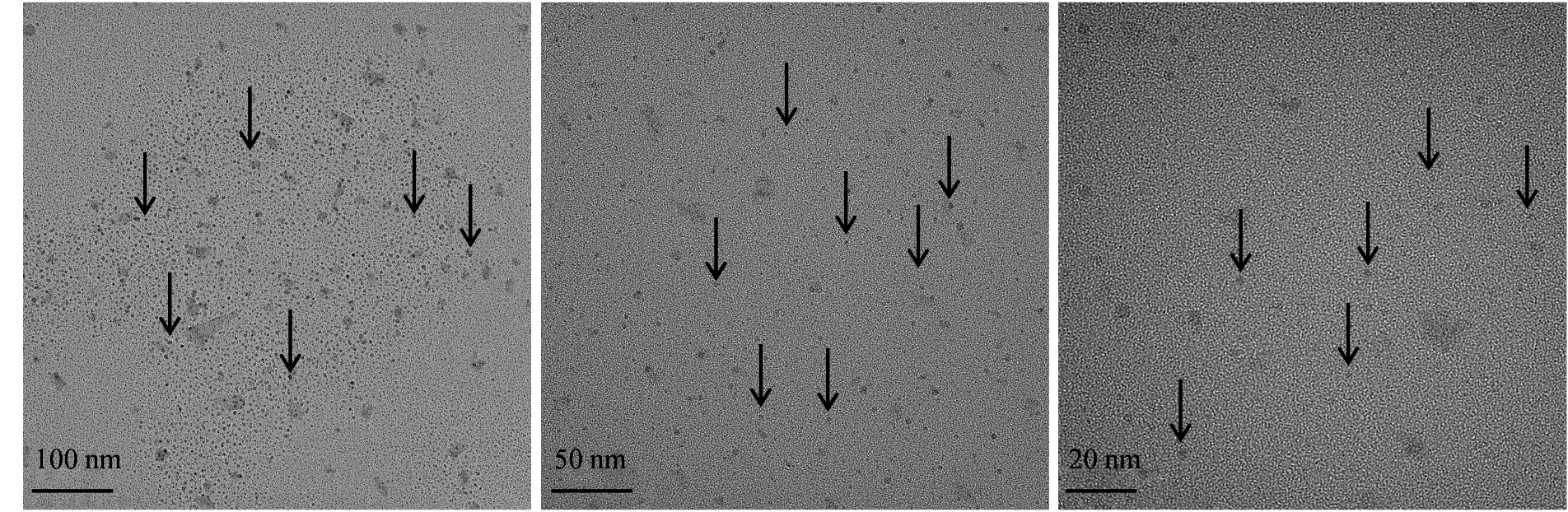


**Figure S11.** The HRETM images of the degraded small particles RVLu under pH =6.5 buffer for 12 h at different magnifications. The black arrow points to the degraded small particles.


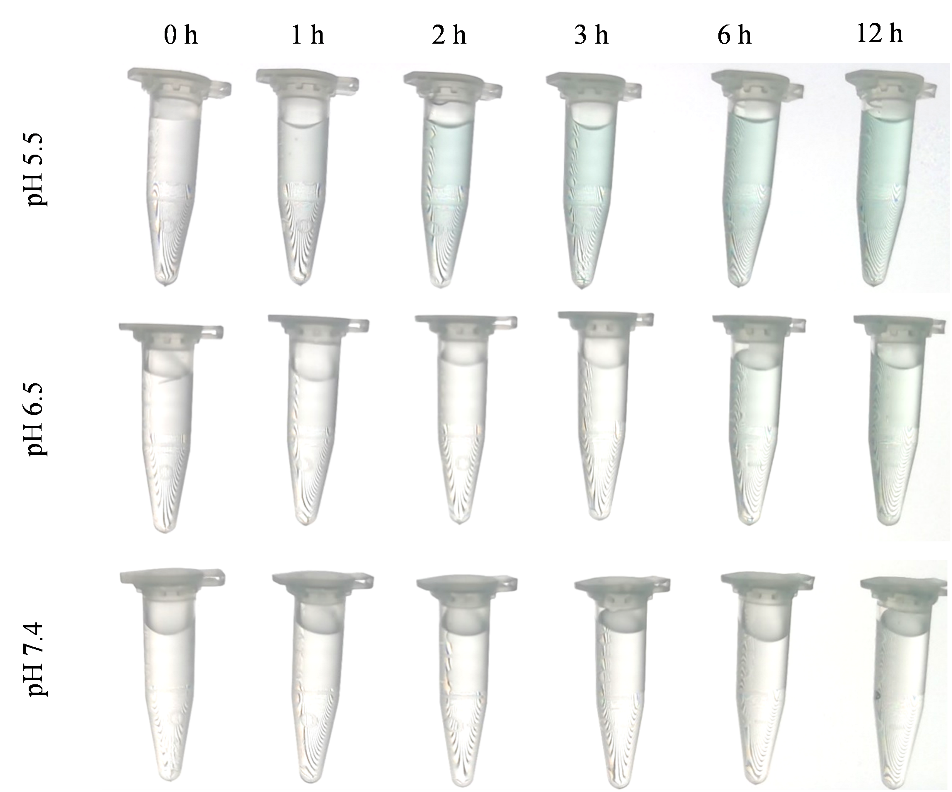
**Figure S12.** White light images of the supernatant of RVLu@ICG incubated with different pH values (7.4, 6.5 and 5.5) for different durations.


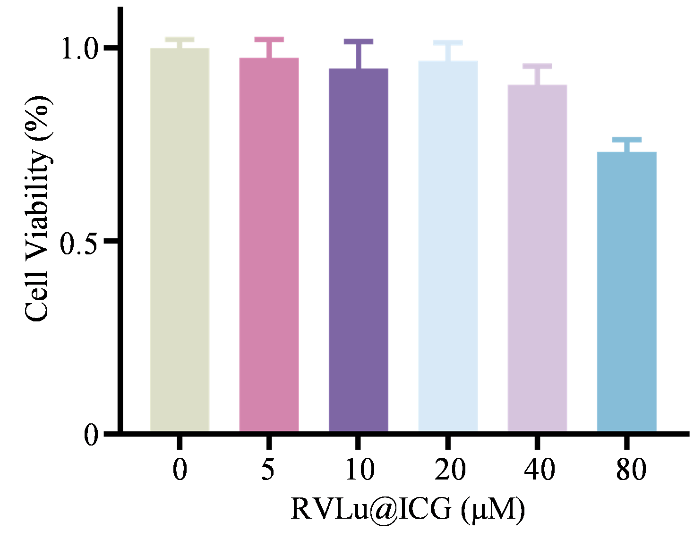
**Figure S13.** Viability of CT26 cells treated with RVLu@ICG at various concentrations.


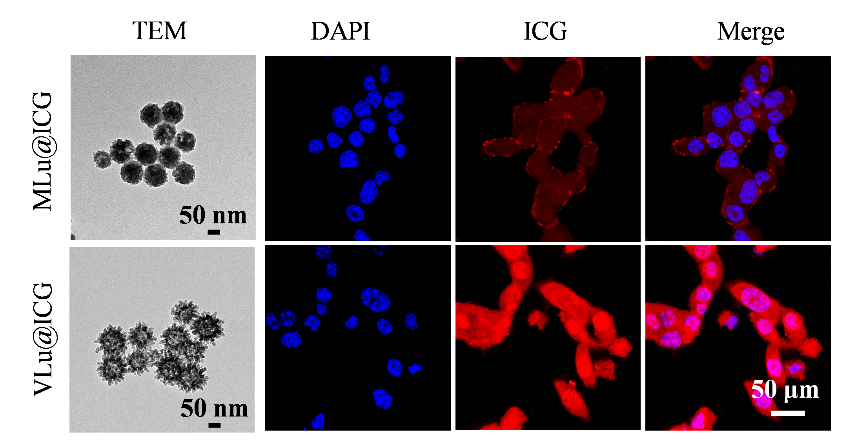
**Figure S14.** Representative CLSM images of CT26 cells after incubation with MLu@ICG or VLu@ICG. CLSM: confocal laser scanning microscope. The scale bar represents 50 µm.


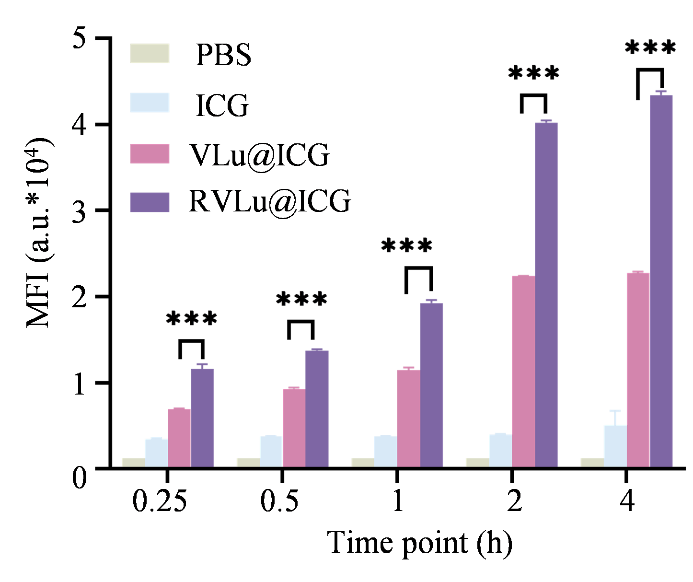
 **Figure S15.** The corresponding MFI of different groups in **Figure 3B**.


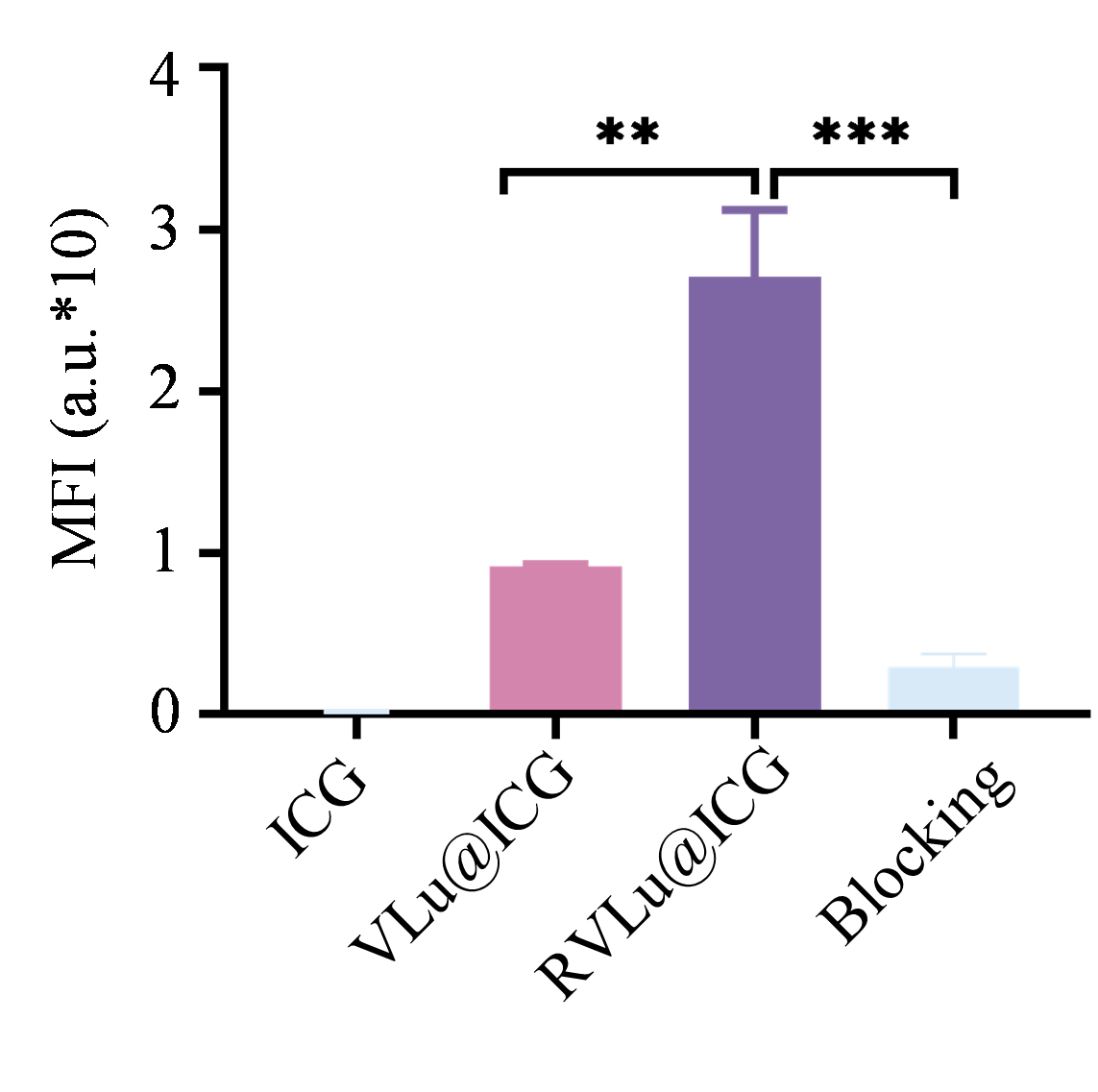
 **Figure S16.** The corresponding MFI of different groups in **Figure 3C**.


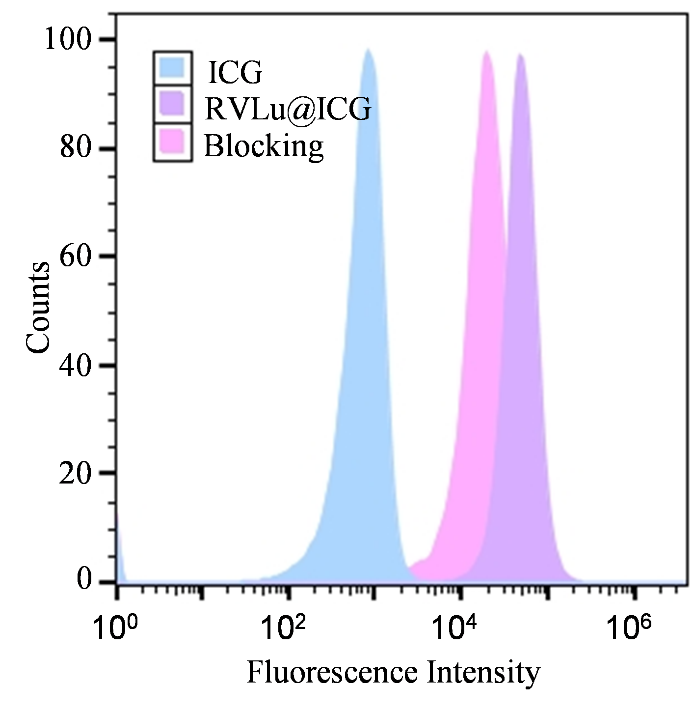
**Figure S17.** Flow cytometry analysis of CT26 cells after incubation with ICG, RVLu@ICG, and RVLu@ICG with c(RGDfK) pretreatment for 1.5 h.


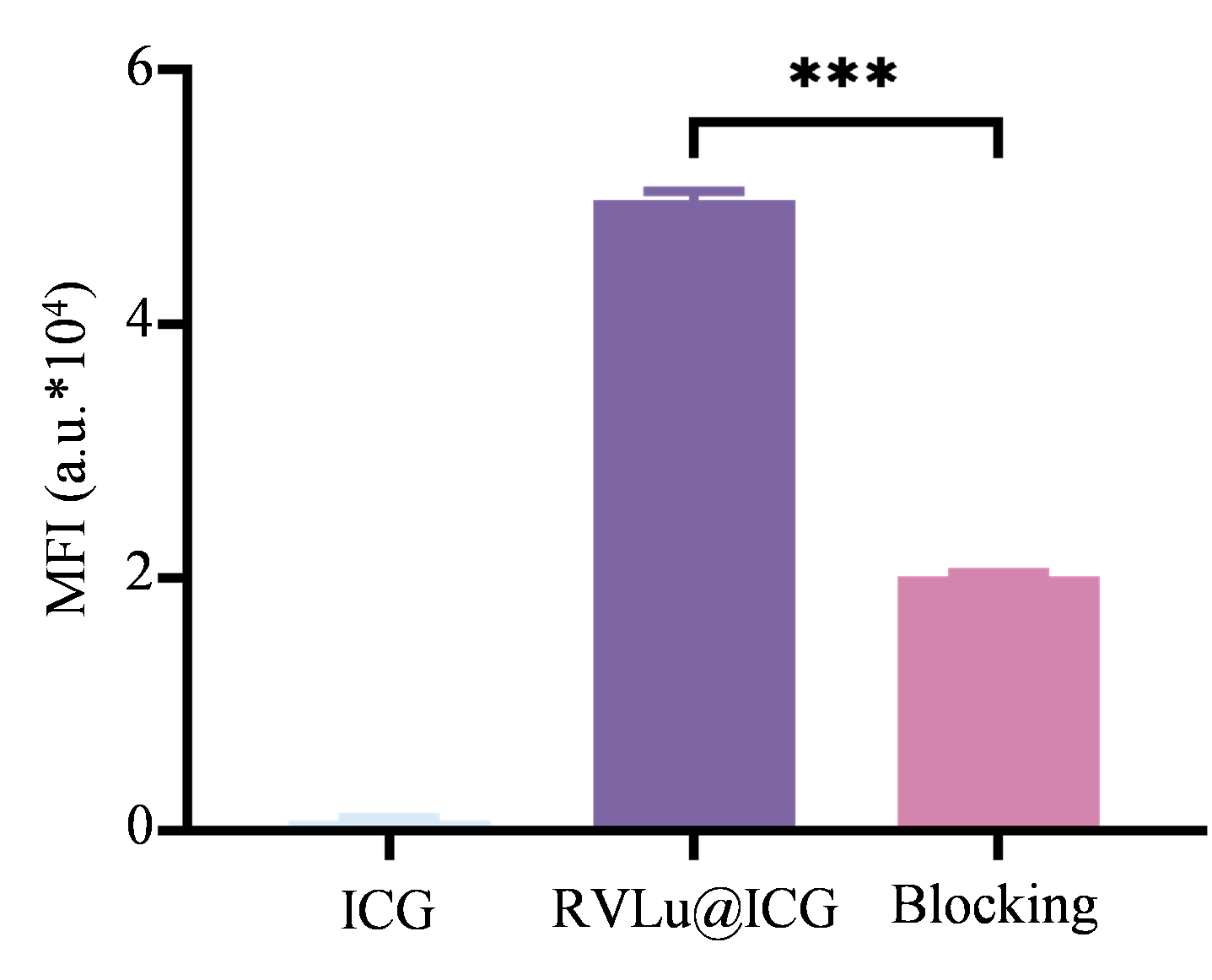
 **Figure S18.** The corresponding MFI of different groups in **Figure S17**.


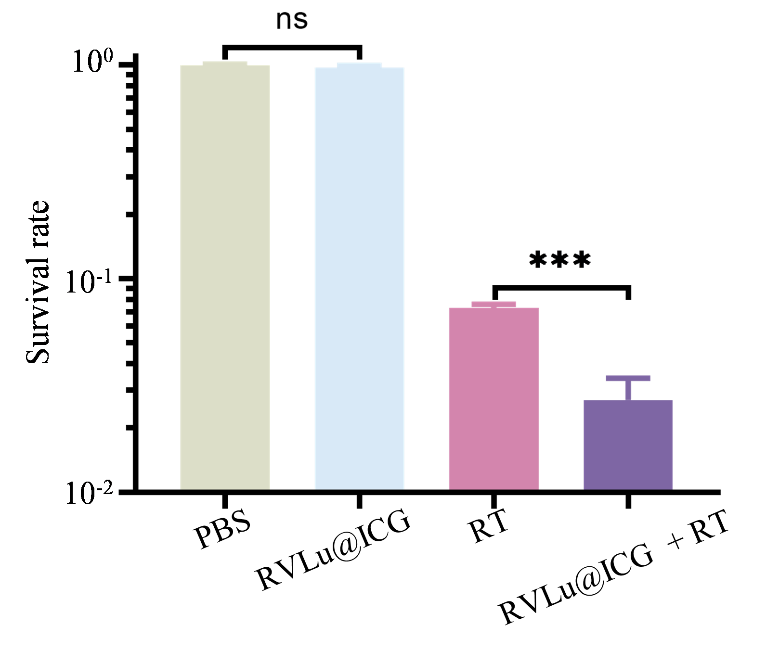
 **Figure S19.** The quantitative analysis of cloning forming efficacy in **Figure 3E**.


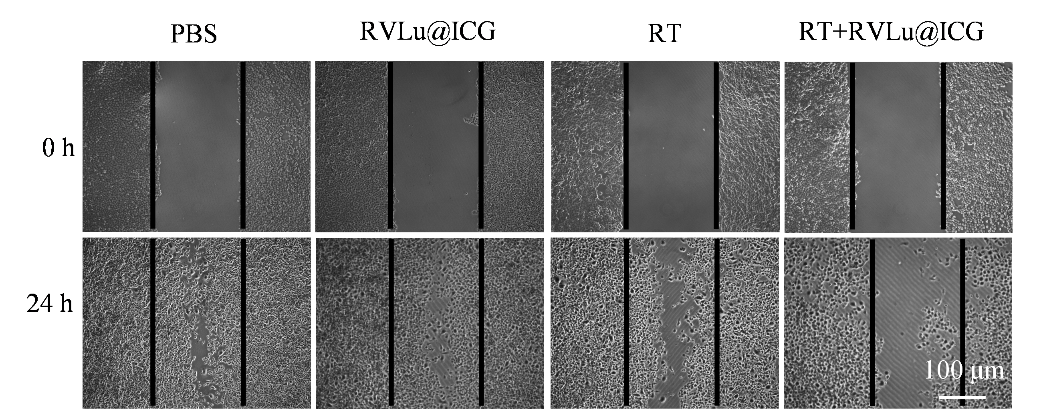
**Figure S20.** Digital images of CT26 cells after the cell scratch treatment. Scale bars: 100 μm.


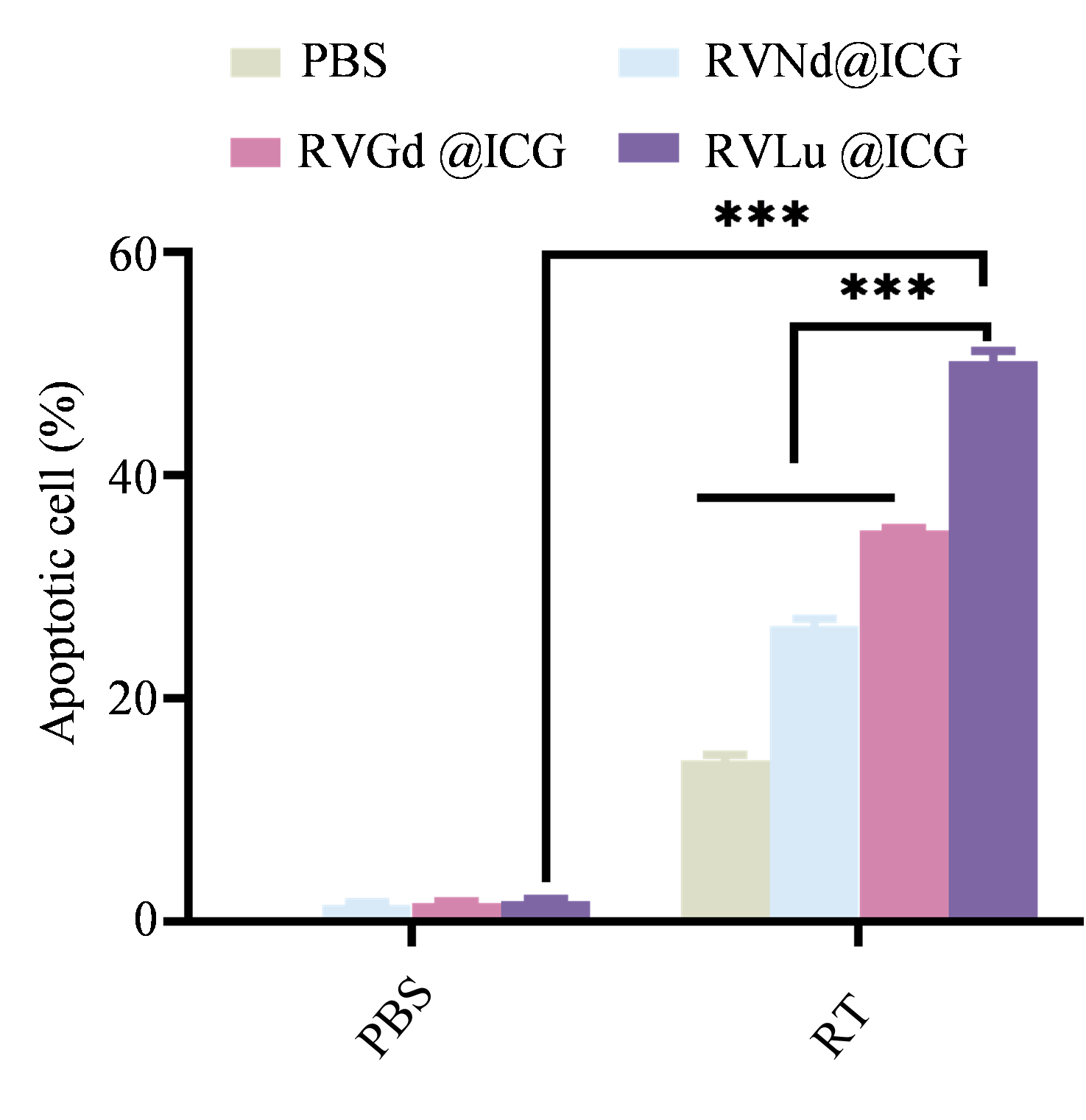
 **Figure S21.** The quantitative analysis of apoptosis rates in **Figure 3F**.

**
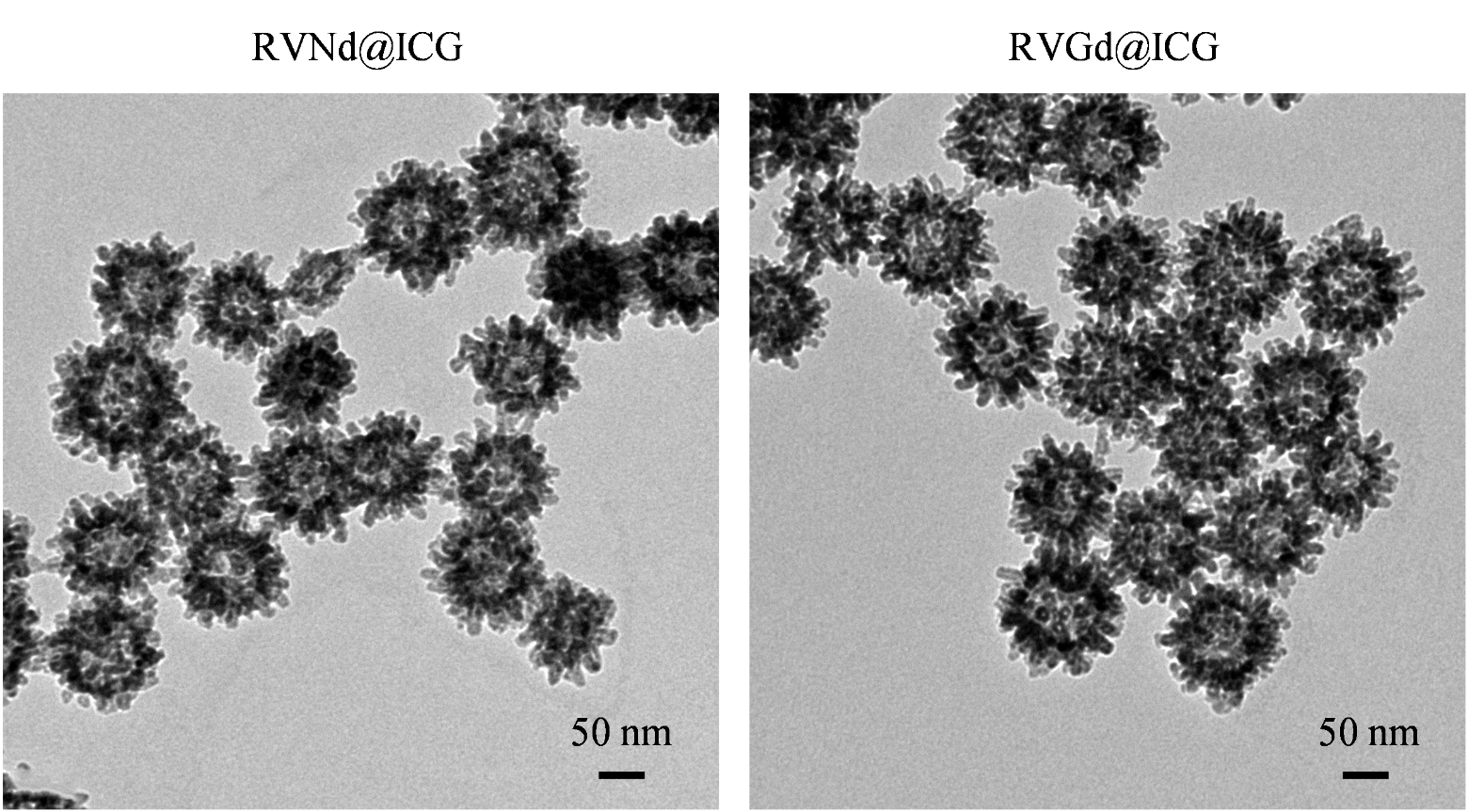
Figure S22.** TEM images of RVNd@ICG and RVGd@ICG.


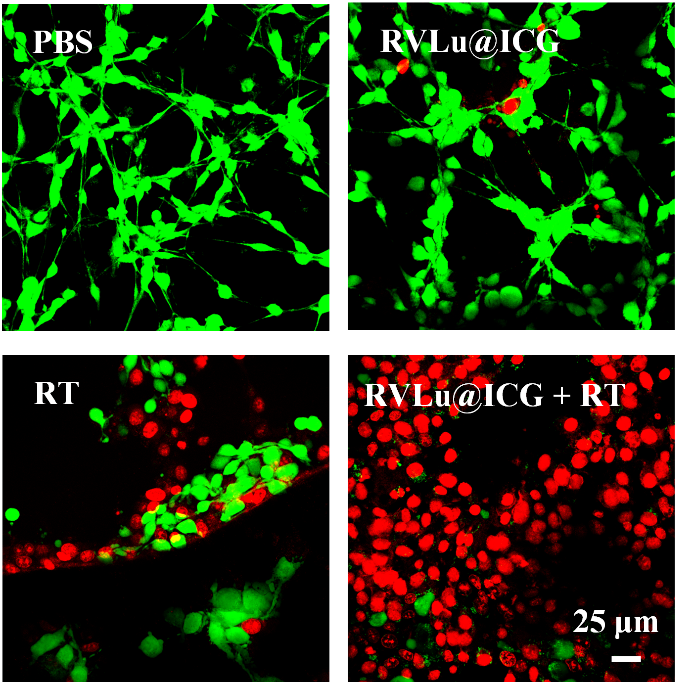
**Figure S23.** CLSM images of live and dead CT26 cells after treated with PBS and RVLu@ICG with or without X-ray irradiation (4 Gy).

**Figure S24.** The bright-field images of the cells
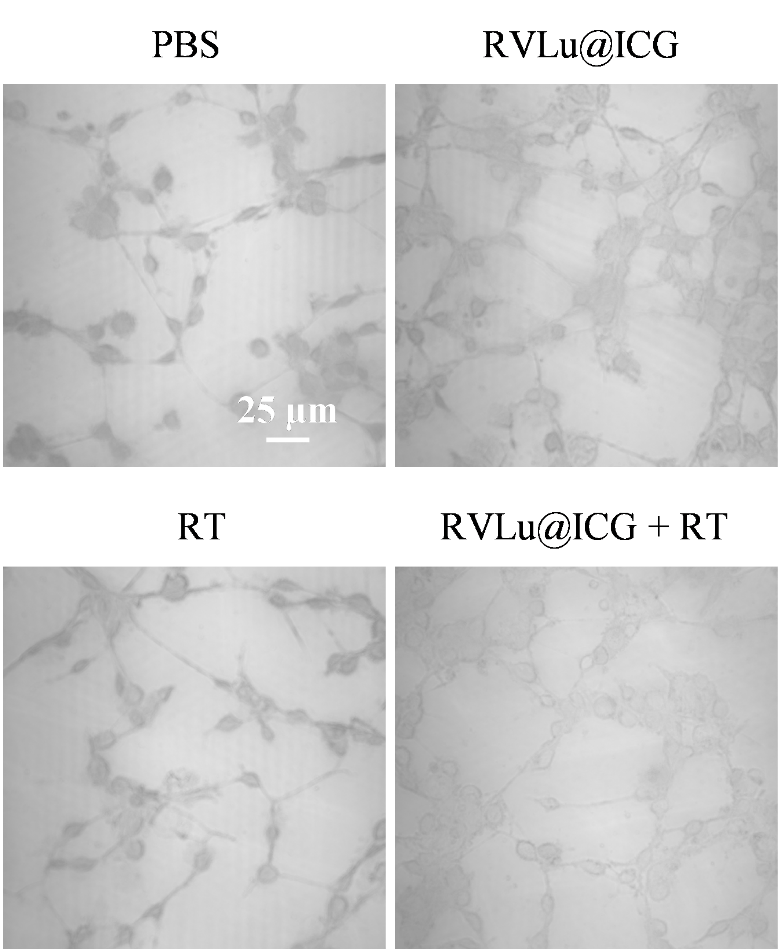
in **Figure 3H**.


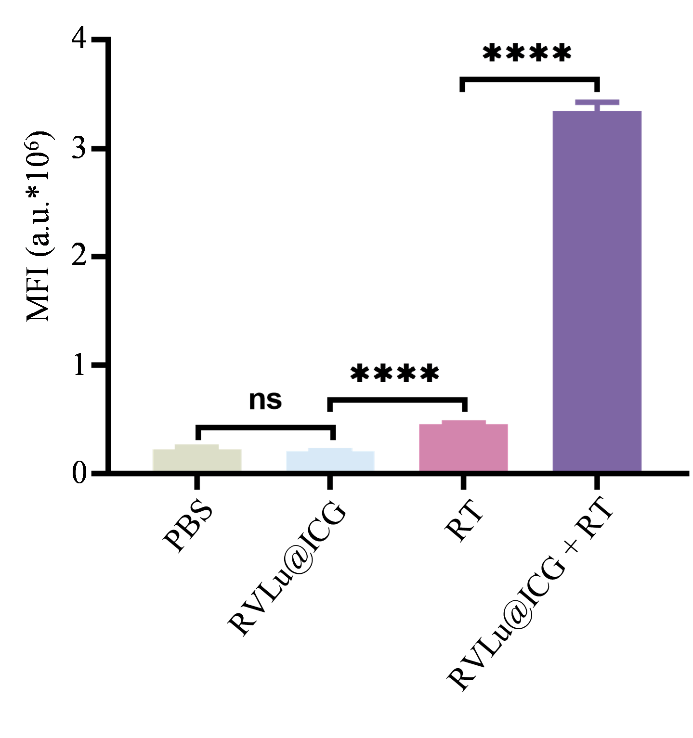
 **Figure S25.** The corresponding MFI of different groups in **Figure 3G**.


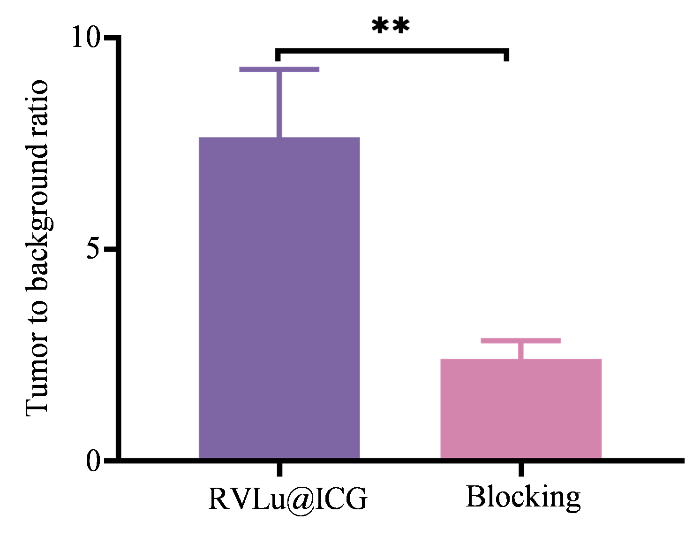
**Figure S26.** Quantitative comparison of TBR between RVLu@ICG targeting group and the c(RGDfK) blocking group (40 mg/kg) 48 h after injection.


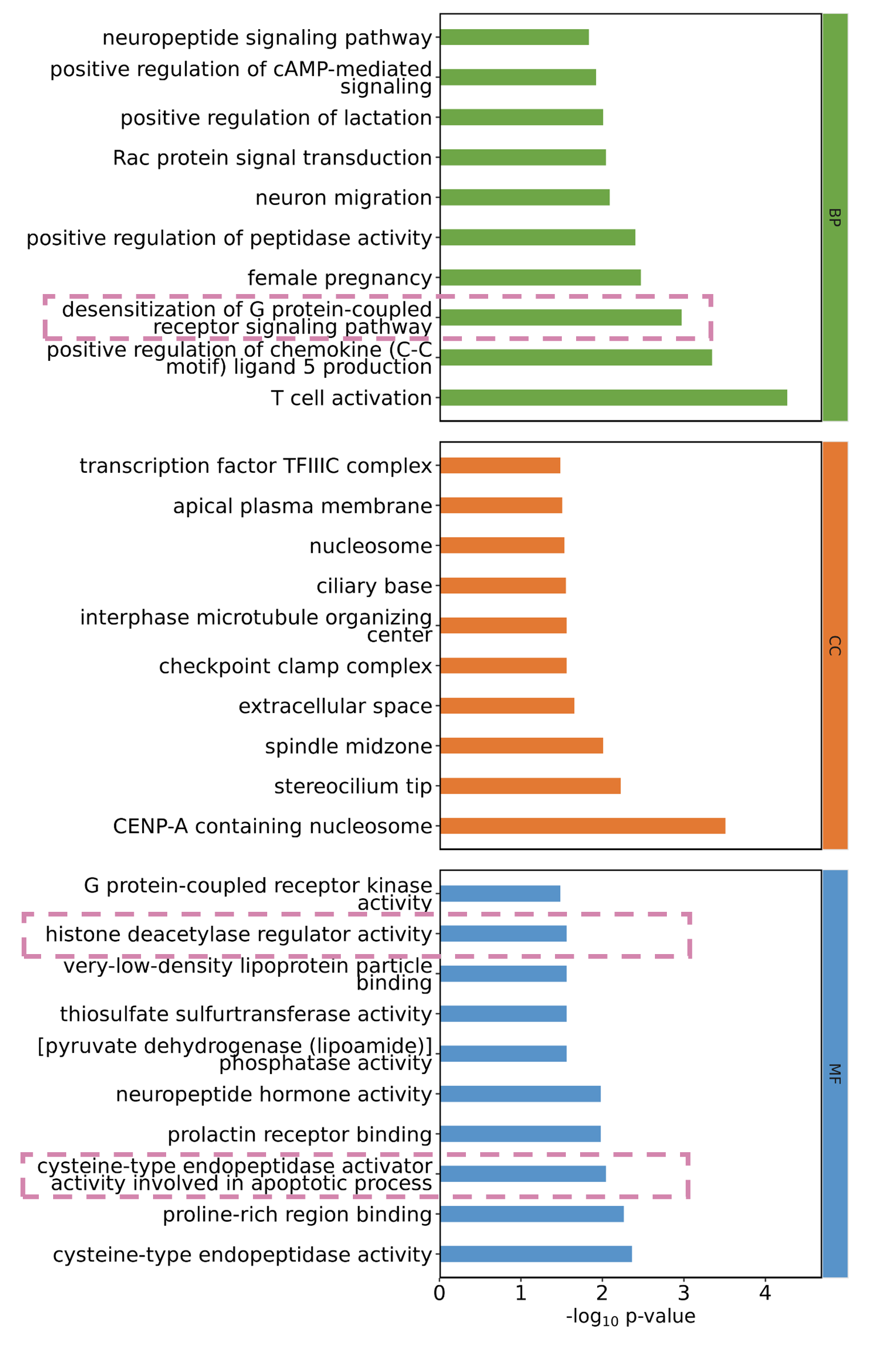
 **Figure S27.** Gene Ontology (GO) enrichment analysis of tumor tissues after receiving different treatments.


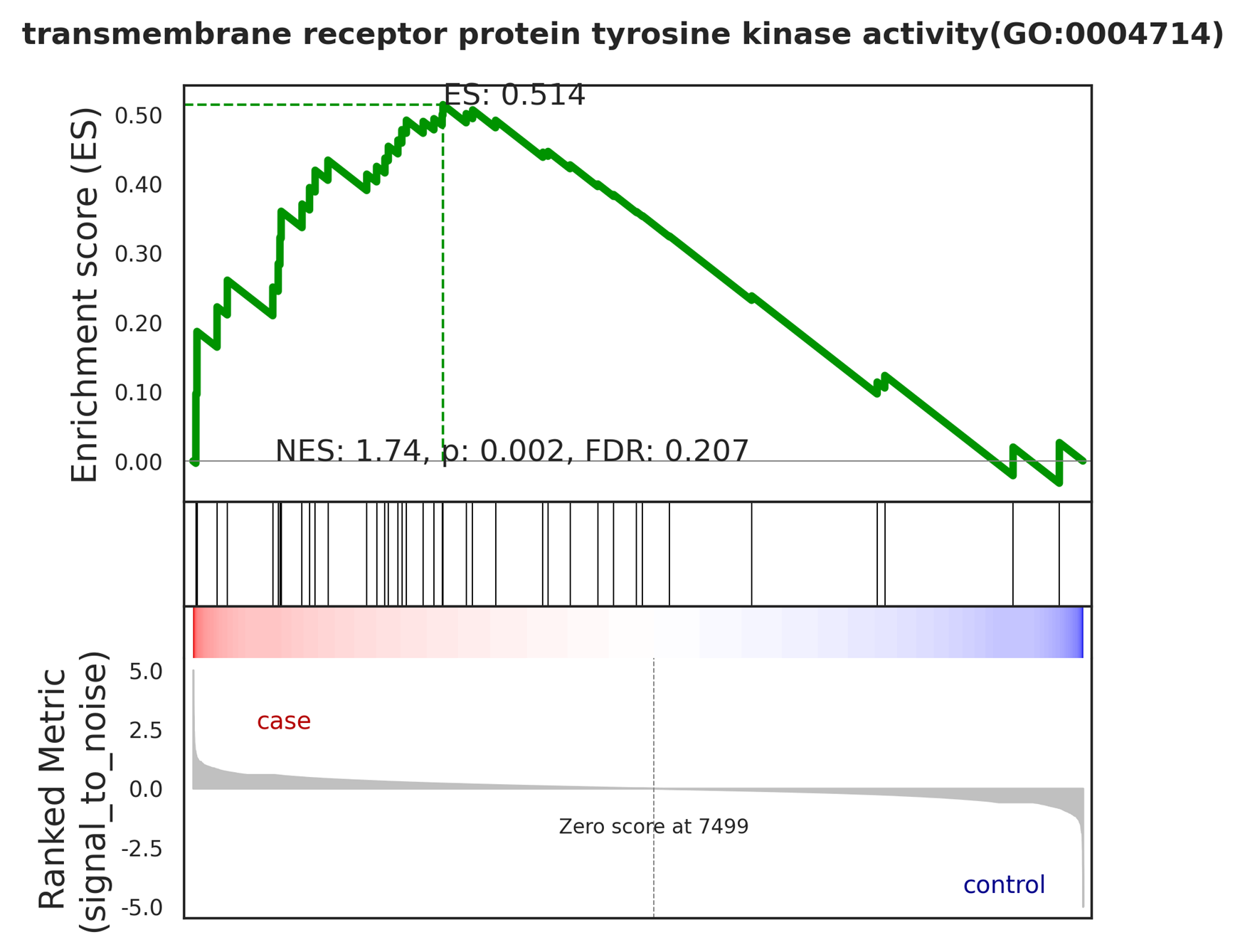
 **Figure S28.** Mountain plot of transmembrane receptor protein tyrosine kinase activity pathway after receiving different treatments.


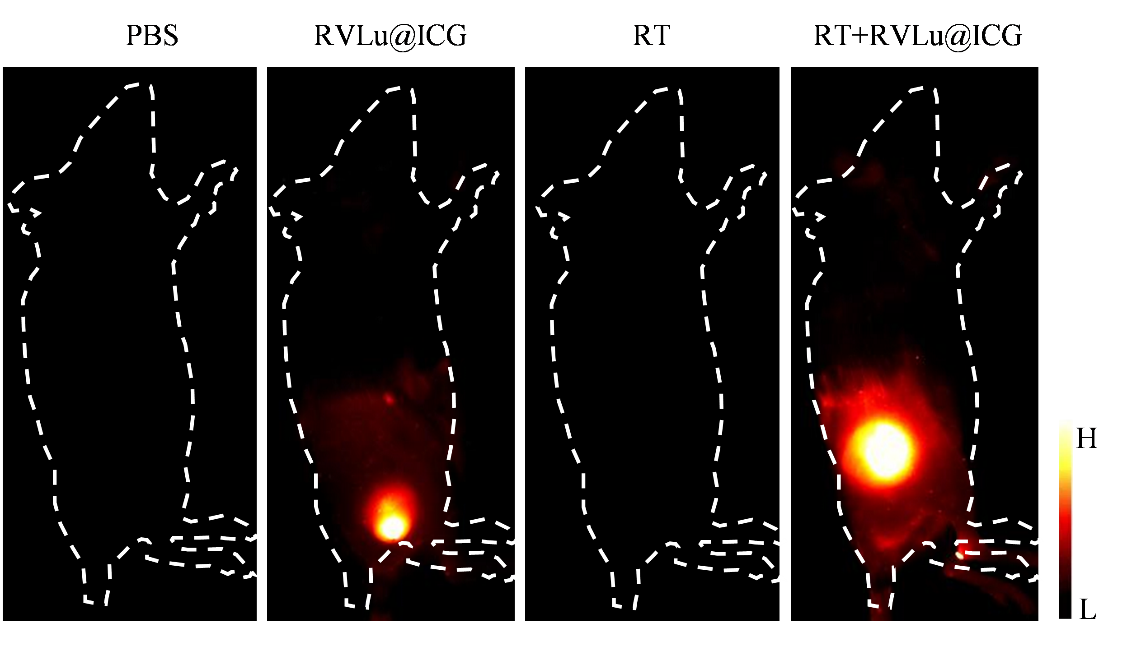
**Figure S29.** Representative images showing fluorescence in mice before treatment with RVLu@ICG and PBS, with or without X-ray radiation therapy.


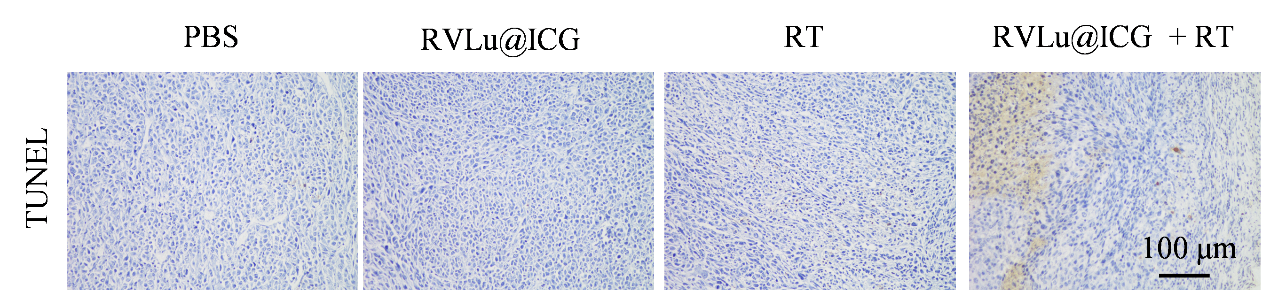


**Figure S30.** TUNEL staining images of tumor after treated with PBS, RVLu@ICG, RT and RVLu@ICG + RT (n = 3). The scale bar is 100 μm.


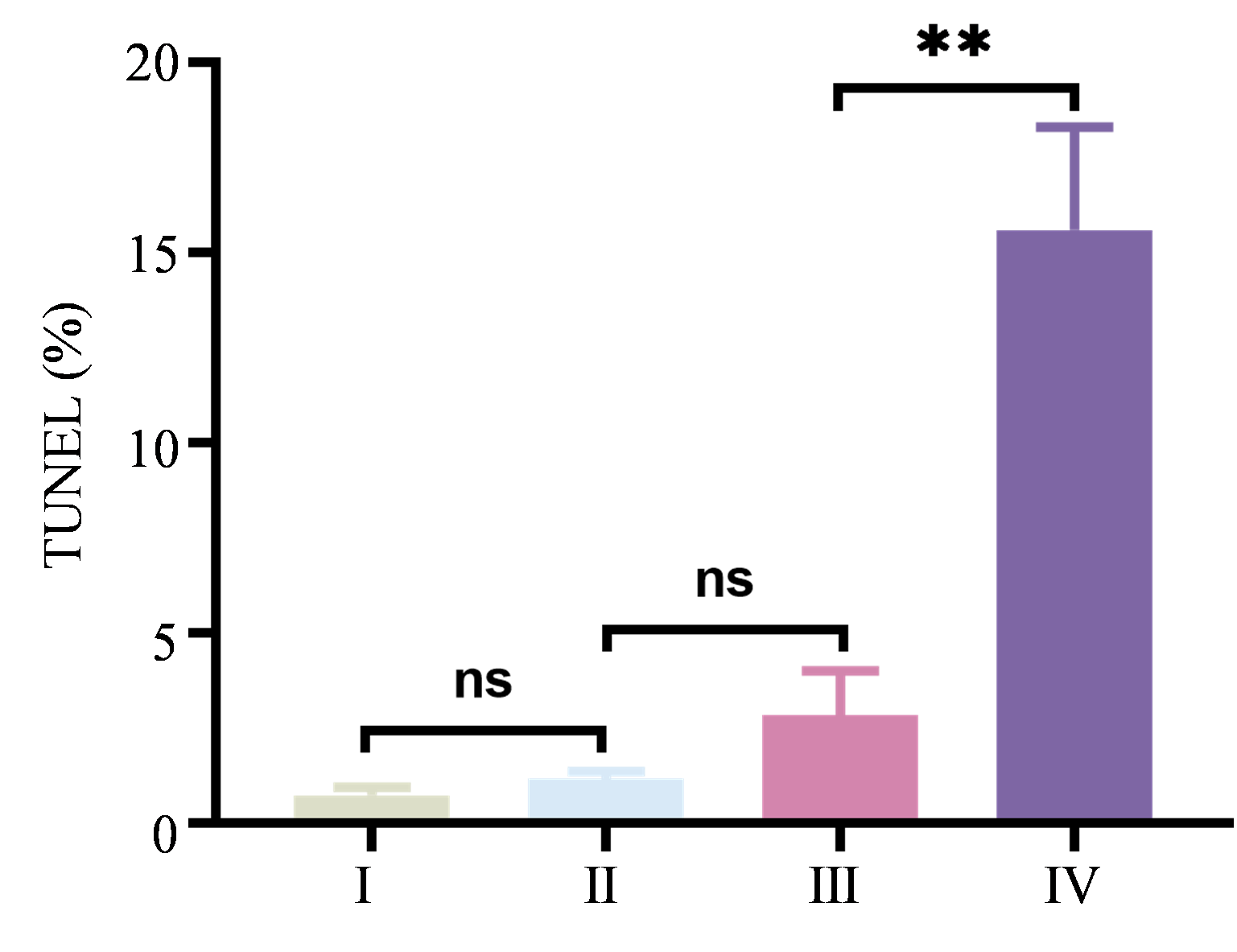
**Figure S31.** The corresponding quantitative analysis of the TUNEL staining in **Figure S30**.


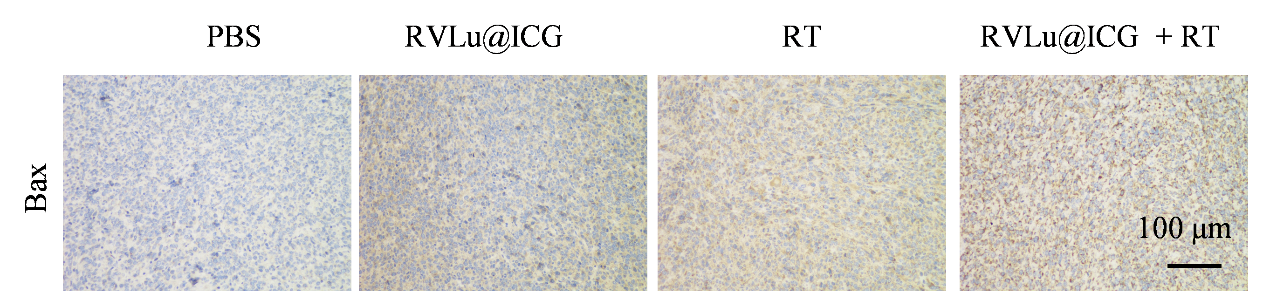
 **Figure S32.** Bax staining images of tumor after treated with PBS, RVLu@ICG, RT and RVLu@ICG + RT. The scale bar is 100 μm.


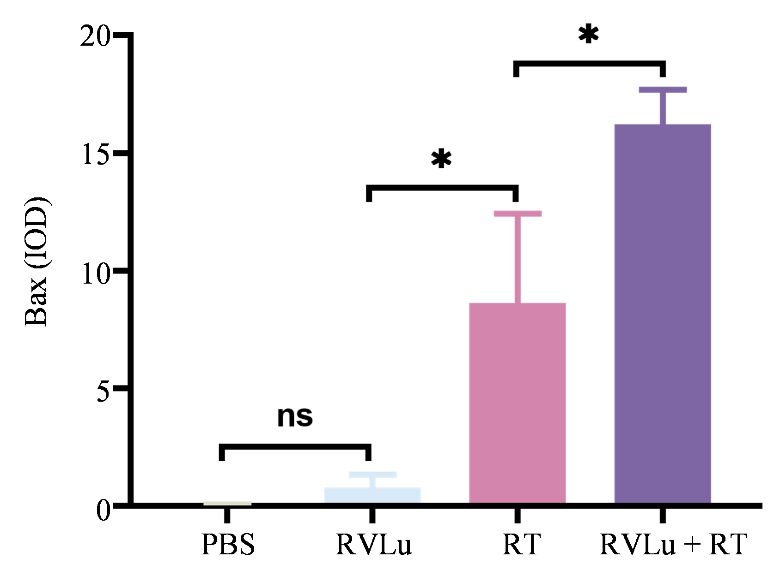
**Figure S33.** The corresponding quantitative analysis of the Bax staining in **Figure S32**.


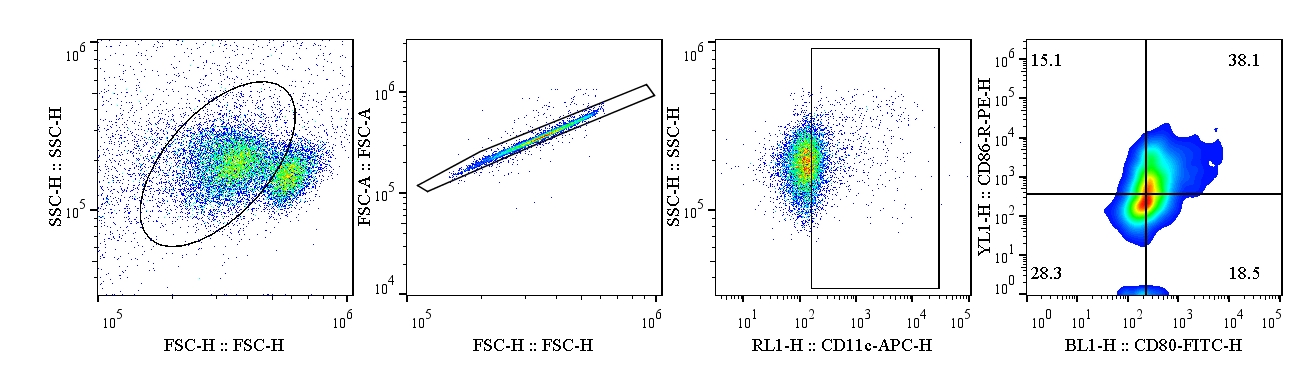


**Figure S34.** Gating strategy to sort matured DCs in tumor-draining lymph nodes.


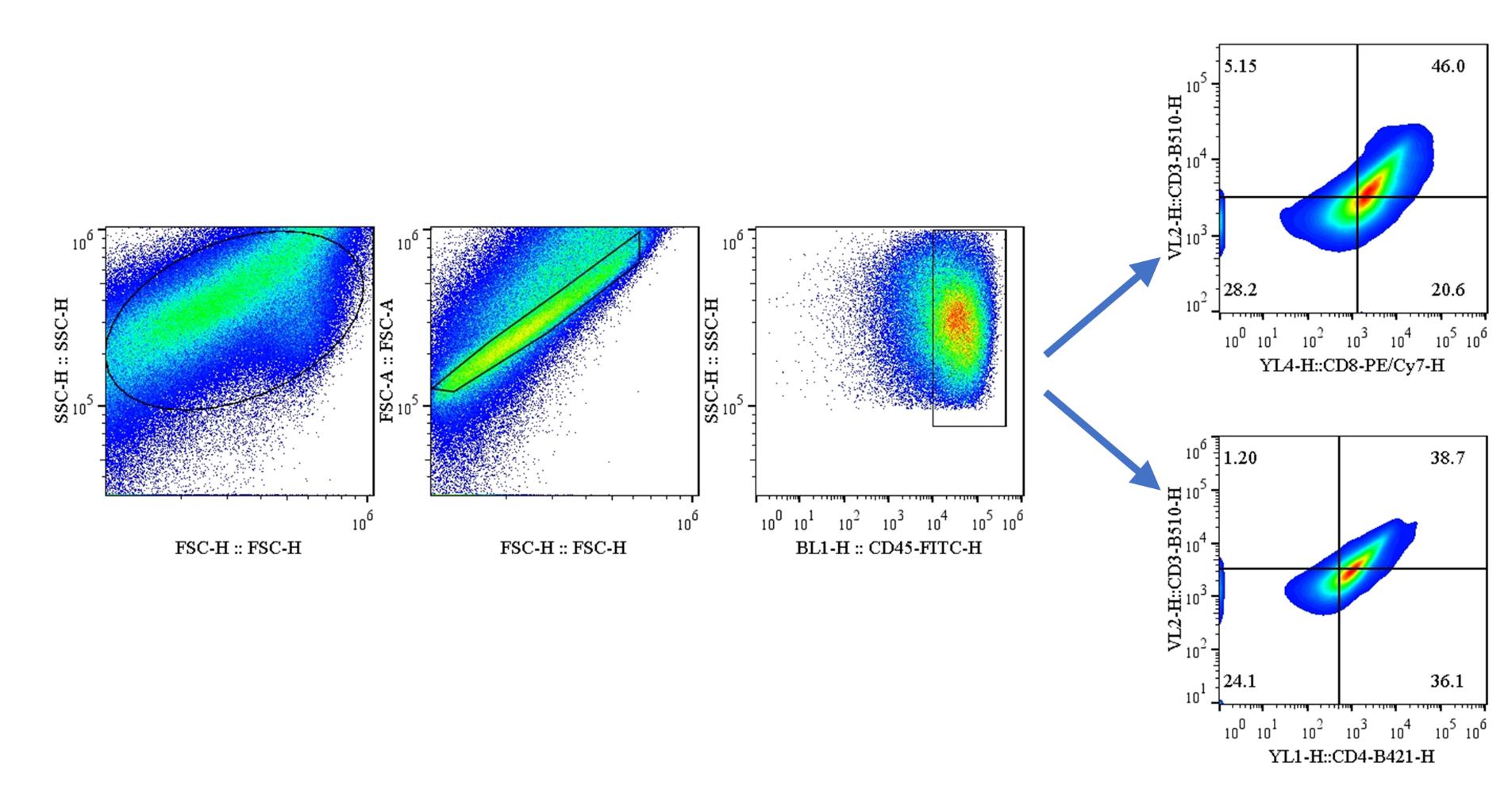

**Figure S35.** Gating strategy to sort CD8^+^ T cells and CD4^+^ T cells in treated tumors.


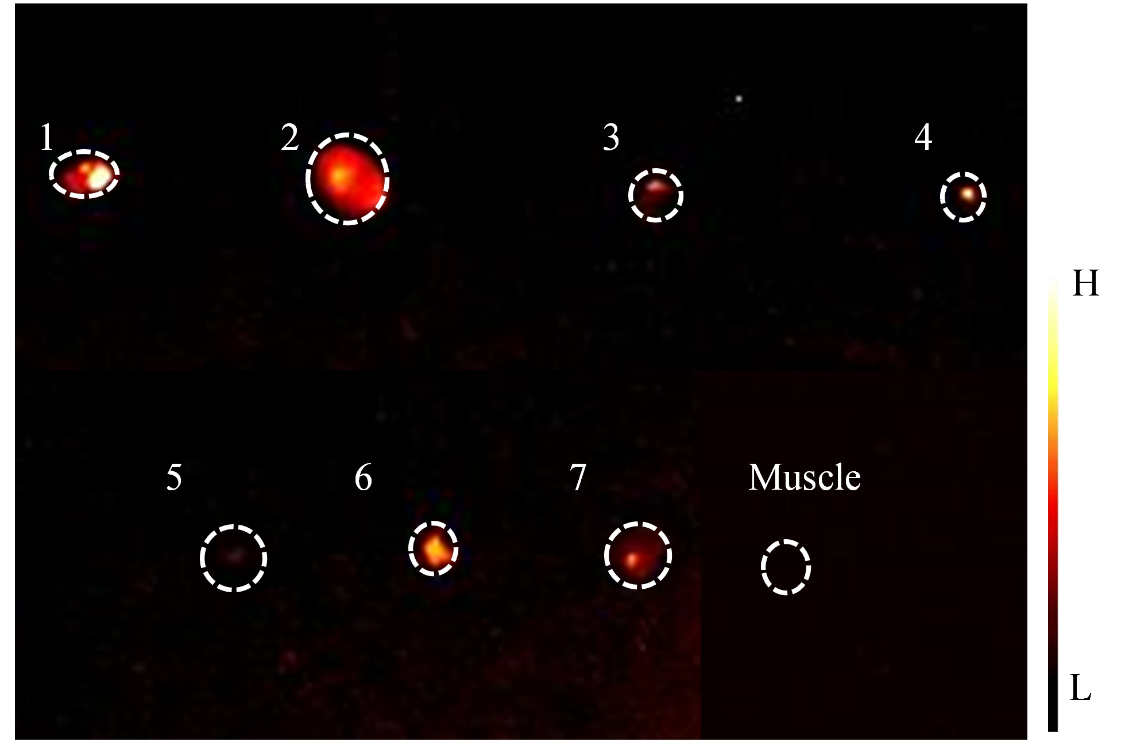
**Figure S36.** NIR-II fluorescence images of resected tissues and a piece of muscle in **Figure 8A**.


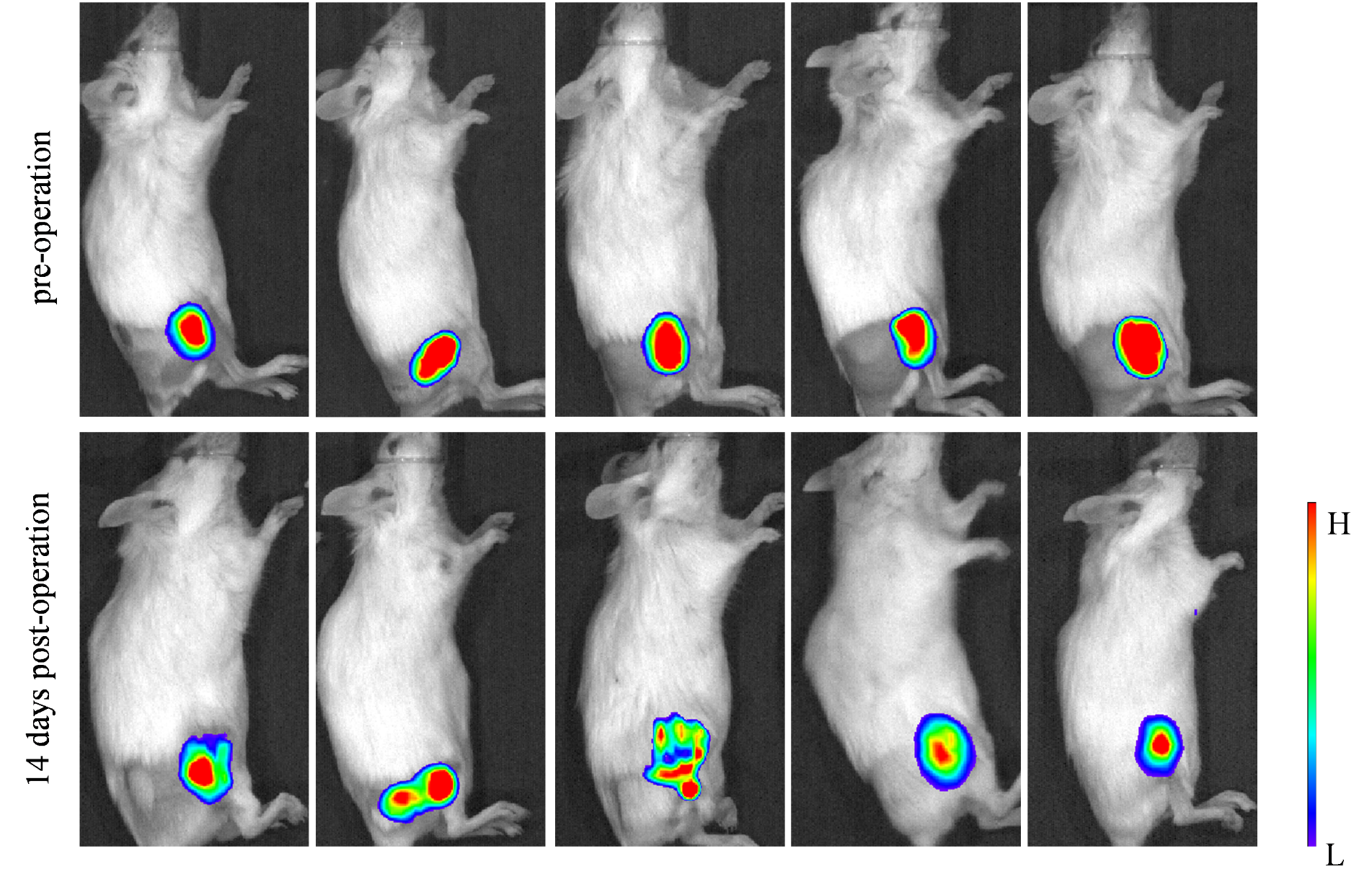
 **Figure S37.** Pre-operation (up) and 14 days post-operation (down) bioluminescence images of the CT26 tumor bearing mice under white light guidance of tumor resection surgery after injected with PBS.


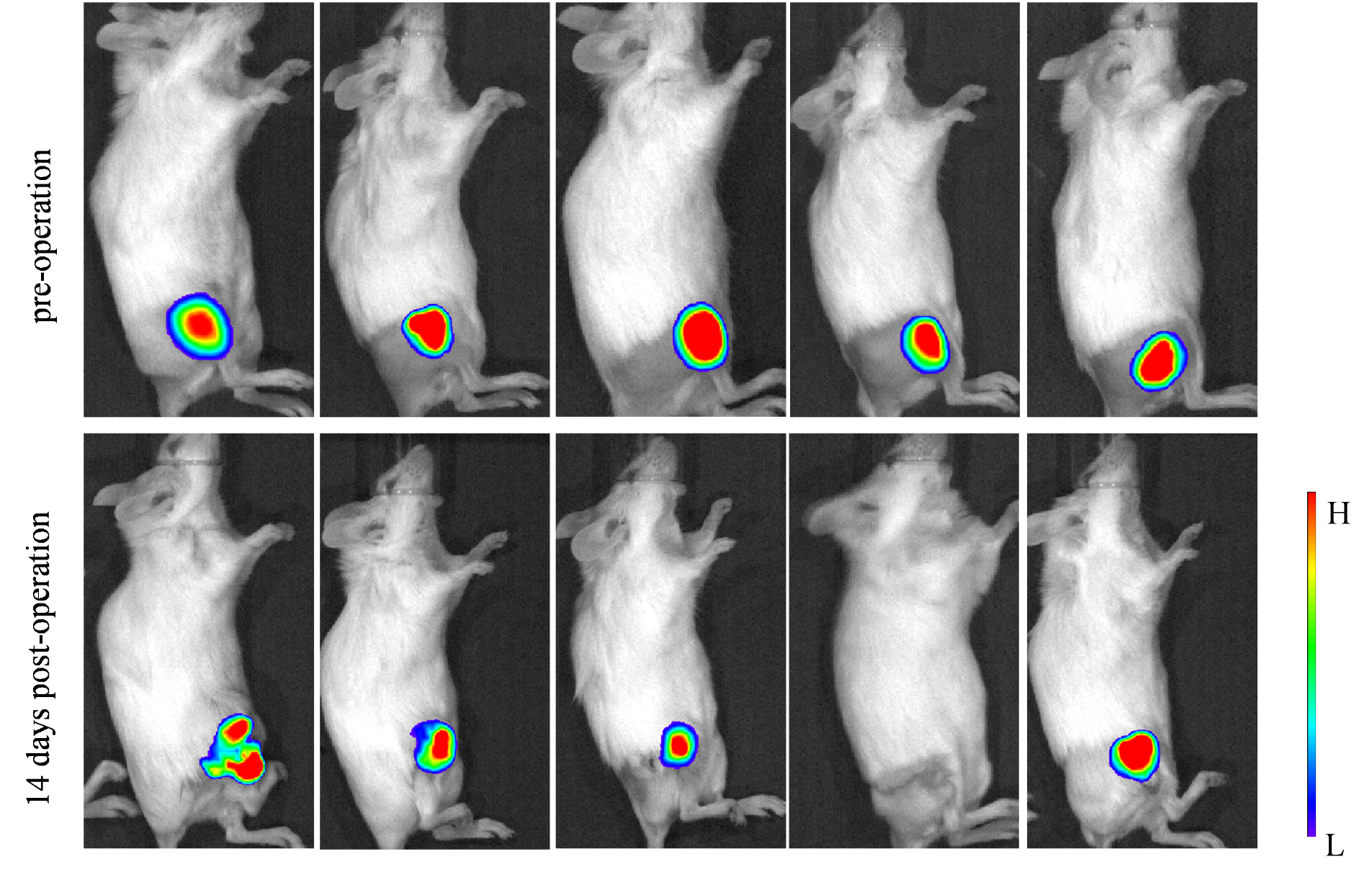
 **Figure S38.** Pre-operation (up) and 14 days post-operation (down) bioluminescence images of the CT26 tumor bearing mice under NIR-II fluorescence guidance of tumor resection surgery after injected with ICG.


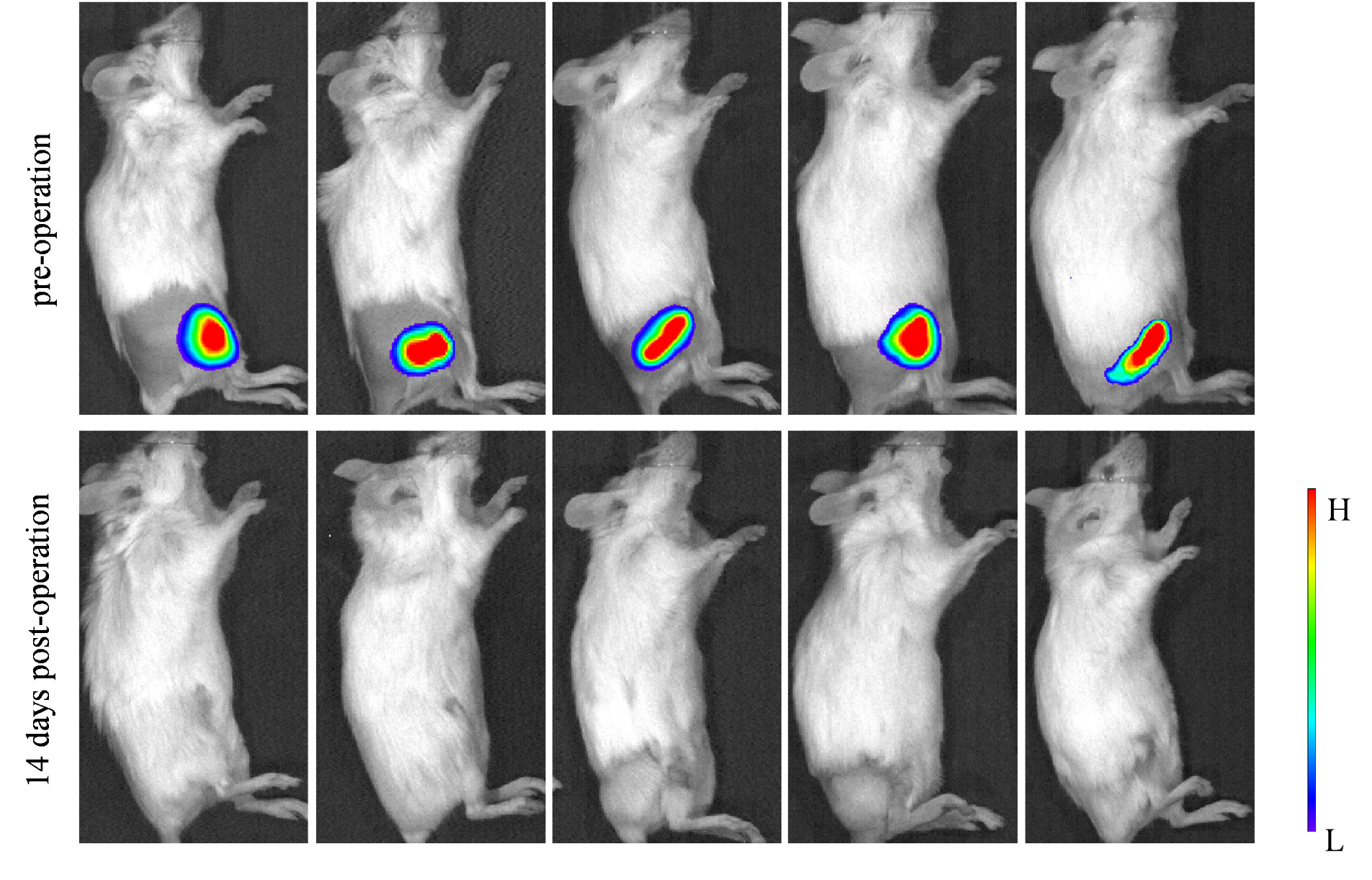
 **Figure S39.** Pre-operation (up) and 14 days post-operation (down) bioluminescence images of the CT26 tumor bearing mice under NIR-II fluorescence guidance of tumor resection surgery after injected with RVLu@ICG.


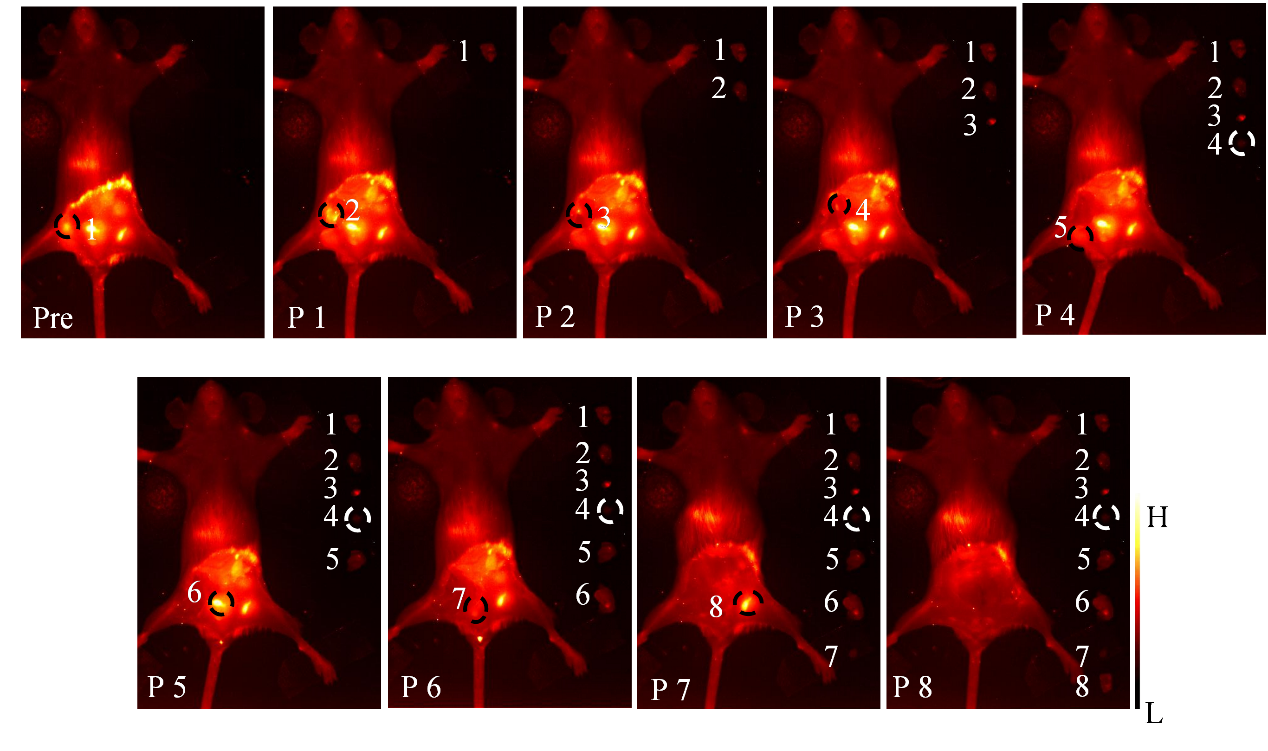
 **Figure S40.** The representative process of step-wise excision of abdominal transfered carcinoma (1-8, the black dotted ellipses) in mice with the guidance of NIR-II fluorescence.


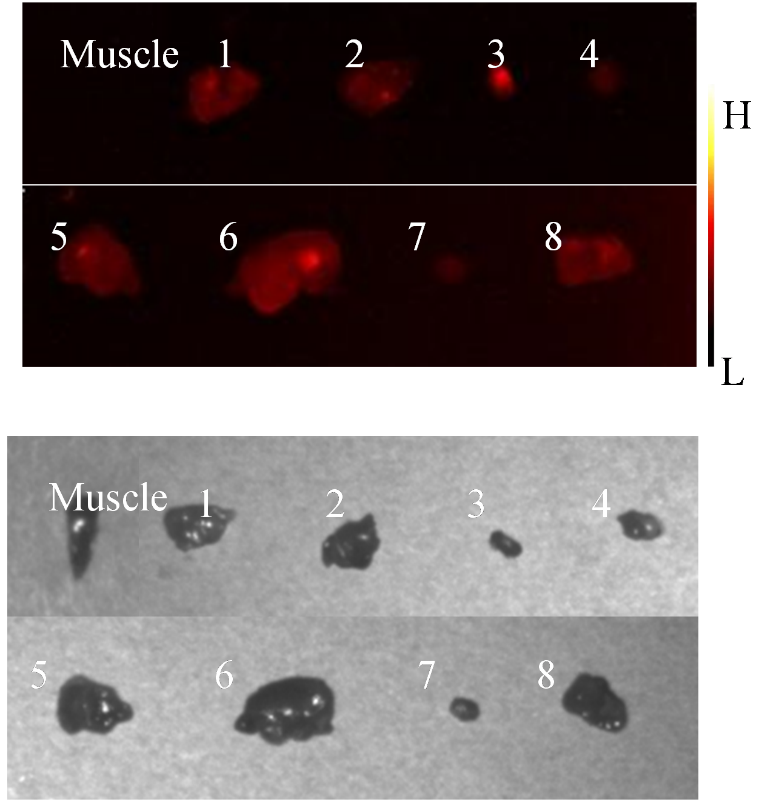
**Figure S41.** NIR-II fluorescence (up) and bright field (down) images of resected tissues and a piece of muscle in **Figure S40**.


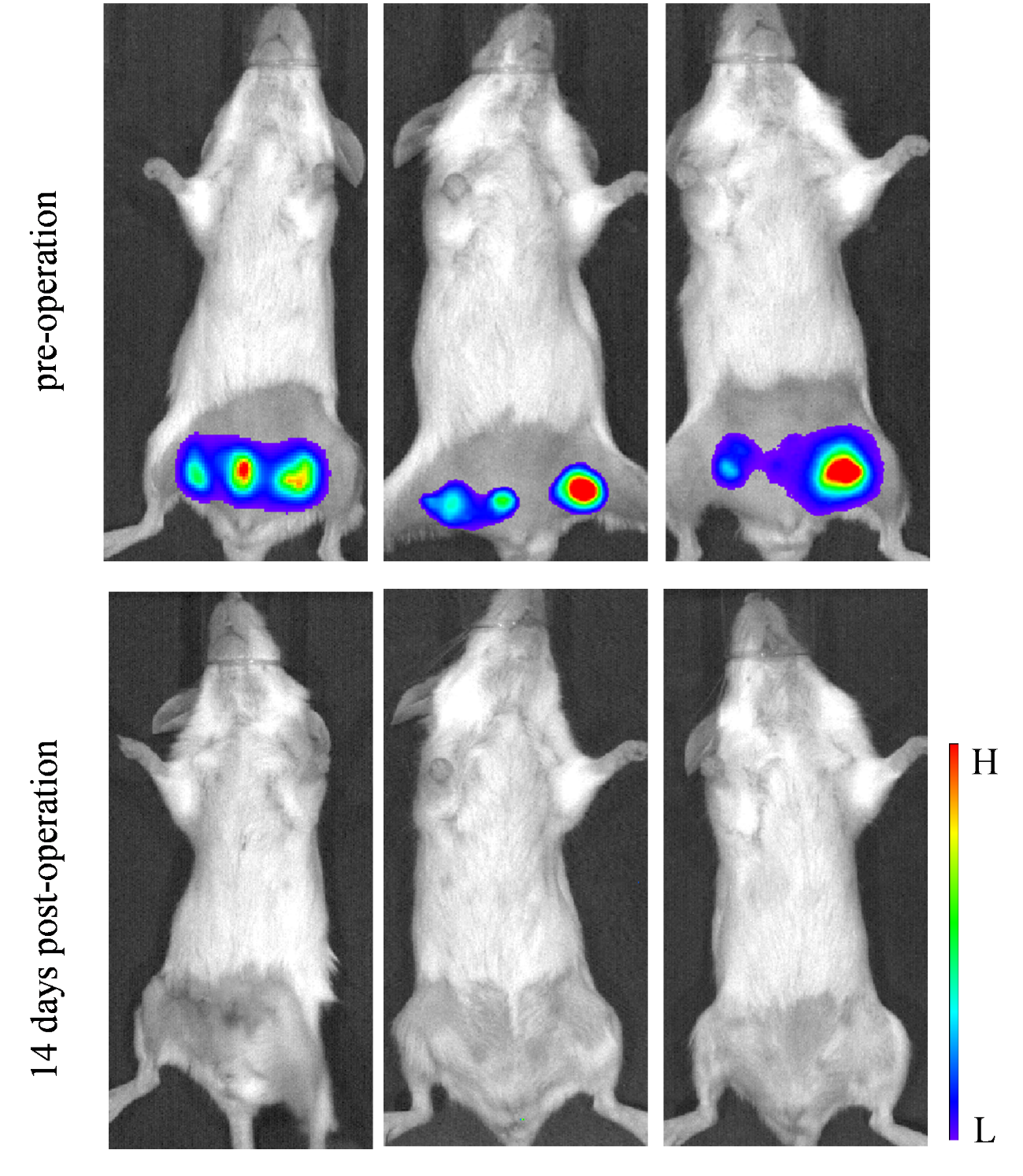
**Figure S42.** The bioluminescence images of the abdominal transfered carcinoma in mice from pre-operation (up) and 14 days post-operation (down) after tumor movement under NIR-II fluorescence guidance.
